# hsa-miR-206b Involves in the Development of Papillary Thyroid Carcinoma via Targeting LMX1B

**DOI:** 10.1155/2022/7488708

**Published:** 2022-03-15

**Authors:** Hongsheng Lu, Chumeng Zhu, Yanyun Ruan, Lilong Fan, Zhengying Ruan, Qi Chen, Jiuyun Yuan, Yang Xu, Hongwei Wang, Qing Wei

**Affiliations:** ^1^Department of Pathology, Shanghai Tenth People's Hospital, Tongji University School of Medicine, Shanghai 200072, China; ^2^Department of Pathology, Taizhou Central Hospital (Taizhou University Hospital), Taizhou, Zhejiang 318000, China; ^3^Precision Medicine Center, Taizhou Central Hospital (Taizhou University Hospital), Taizhou, Zhejiang 318000, China; ^4^Nanjing Geneseeq Technology Inc., Nanjing, Jiangsu, China; ^5^Department of Pathology, People's Hospital, The Affiliated Hospital of Ningbo University, Ningbo, China

## Abstract

**Objectives:**

Papillary thyroid carcinoma (PTC) is the most common endocrine system malignant thyroid cancer, and patients with lymph node metastasis typically exhibit poor prognosis. MicroRNAs (miRNAs) can act as either oncogenes or tumor suppressors in PTC. This study was aimed at using PTC transcriptome data obtained from The Cancer Genome Atlas (TCGA) to identify differentially expressed, survival-related miRNAs and target genes.

**Methods:**

We analyzed the TCGA datasets to identify differentially expressed mRNAs/miRNAs in 493 PTC patients with stage I_II group (stages I and II) versus stage III_IV group (stages III and IV) according to TNM staging. The Kaplan-Meier survival analysis, the Cox regression analysis, and the log-rank test were performed to investigate survival-related miRNAs.

**Results:**

We identified 36 significantly differentially expressed miRNAs in the stage I_II group versus the stage III_IV group, in which 31 were upregulated and only 5 were downregulated (i.e., hsa-miR-891a-5p, hsa-miR-892a, hsa-miR-888-5p, hsa-miR-891b, and hsa-miR-892b). Additionally, five signature miRNAs (hsa-miR-206, hsa-miR-299-3p, hsa-miR-299-5p, hsa-miR-496, and hsa-miR-509-3-5p) were associated with the overall survival of PTC patients. We also found that *LMX1B*, whose expression was inversely correlated with hsa-miR-206 expression, was a putative target gene of hsa-miR-206 and *LMX1B* was likely to serve as a tumor suppressor in PTC.

**Conclusion:**

hsa-miR-206b might be involved in promoting TNM staging in PTC via targeting of *LMX1B*.

## 1. Introduction

MicroRNAs (miRNAs) are endogenous single-stranded noncoding RNAs with a length of 19-24 nucleotides. miRNAs have been used as biomarkers for various cancers, and they can function as either oncogenes or tumor suppressors depending on their specific targeted genes [1, 2]. For example, miRNA-435-5p [3] and miRNA-302s [4] have been shown to act as oncogenes in colorectal cancer and testicular germ cell tumor, respectively. On the other hand, the miR-200 family functioned as putative tumor suppressors and was usually downregulated in human cancer cells [5]. Therefore, the elucidation of functions of various miRNAs in certain cancerous contexts is still the key to understanding the mechanism of tumorigenesis and disease progression.

Thyroid cancer is the most common endocrine system malignant tumor in China with around 756,000 newly diagnosed cases and nearly 52,000 deaths per year [6, 7]. Papillary thyroid carcinoma (PTC) is the most frequent subtype of thyroid cancer, accounting for <80% of cases [8–10]. Currently, surgical resection is mainly considered as a curative therapy for thyroid carcinoma; however, patients with lymph node metastasis typically exhibit poor prognoses. Some previous studies explored the potential use of miRNAs as diagnostic and prognostic tools in thyroid cancers. Specifically, a broad range of miRNAs in PTC, such as miR-146b-3p, miR-146-5p, miR-221, miR-222, and miR-224, are accompanied by a significant upregulation when compared with those of nonneoplastic thyroid tissues [11–13]. In contrast, the expression level of miR-204, miR-219-5p, miR-451, miR-1179, miR-138, and miR-144-5p was negatively correlated with the growth of PTC cells [11, 14]. In terms of clinical diagnosis, the PTC-upregulated miRNAs are of great interest, as they could potentially improve the accuracy of PTC diagnosis, resulting in over 88% of sensitivity in cancer detection when three or more miRNAs were upregulated. Meanwhile, specifically downregulated miRNA is also important in disease diagnosis. Additionally, the expression of specific miRNA could act as a prognostic factor. For example, overexpression of miR-146-5p, miR-221, and miR-222 and the loss of miR-204 were associated with tumor aggressiveness and progression in thyroid cancer [11].

The emergence of high-throughput sequencing methods has been greatly facilitated the characterization of miRNA and gene expression profiles. Swierniak et al. reported that a total of 427 miRNAs (16.5%) were differentially expressed (>5 reads per million reads) in miRbase in the thyroid gland [14, 15]. The Cancer Genome Atlas (TCGA) also published a large-scale cancer genomics dataset of 507 PTCs and 59 matched normal adjacent tissues from different institutions [8]. In this study, we used mRNA sequencing (mRNAseq) and miRNAseq data from TCGA to identify differentially expressed survival-related miRNAs. The differential expression of miRNAs, combined with their target gene and binding site, may provide a novel understanding of the diagnostic and prognostic function of miRNA in thyroid cancers.

## 2. Materials and Methods

### 2.1. Papillary Thyroid Cancer Data Resource

The mRNA and miRNA transcriptome data of 507 cases of PTC patients were downloaded from TCGA data portal in March 2019 (https://tcga-data.nci.nih.gov/tcga/dataAccessMatrix.htm). The patient's clinical information was obtained from the data transfer tool of the National Cancer Institute Genomic Data Commons (https://gdc.cancer.gov/access-data/gdc-data-transfer-tool). Considering the availability of TNM staging records (stages I, II, III, and IV), the transcriptome data of a total of 493 PTC patients met the requirements for subsequent analysis.

### 2.2. Identification of Differentially Expressed mRNA and miRNA

The “edgeR” (https://bioconductor.org/packages/release/bioc/html/edgeR.html) was used to perform differential analysis between the stage I_II group (stages I and II) and the stage III_IV group (stages III and IV). Genes that met the criteria of ∣fold change | >2 and adjusted *P* value < 0.05 were considered to have significant differences. Heatmaps of mRNA and miRNA expression were generated using hierarchical clustering by the “pheatmap” package (http://www.bioconductor.org/packages/release/bioc/html/heatmaps.html), while the volcano plot was generated to display mRNA and miRNA profiles by the “ggplot” package (https://mirrors.tuna.tsinghua.edu.cn/CRAN/web/packages/ggplot2/index.html). ENSEMBL (htps://http://www.ensembl.org/) was used to annotate the differentially expressed mRNAs and miRNAs.

### 2.3. Univariate Cox Regression and Survival Analysis

Differentially expressed miRNAs were selected with the univariate Cox regression analysis to identify prognosis-associated miRNAs. According to the prognosis-associated miRNA expression value of each sample, patients were divided into the high-expression group and low-expression group with the median value as the threshold. The Kaplan-Meier analysis was used to evaluate the survival of the two groups. The two analysis was performed by the “survival” package (https://mirrors.tuna.tsinghua.edu.cn/CRAN/web/packages/survival/index.html) and the “survminer” package (https://mirrors.tuna.tsinghua.edu.cn/CRAN/web/packages/survminer/index.html), with *P* value < 0.05 being considered as statistically significant.

### 2.4. Target Prediction of Key Prognosis-Associated miRNAs

The differentially expressed key miRNAs were selected for target prediction by using miRanda (https://www.miranda.org/) and TargetScan (http://www.targetscan.org/) databases. To improve the accuracy of target prediction, we further combined the analysis of differentially expressed mRNA with target prediction of the differentially expressed key miRNAs. The intersecting gene set was subject to downstream analysis.

### 2.5. KEGG Enrichment Analysis

The KEGG enrichment analysis was performed by the “clusterProfiler” package (https://www.bioconductor.org/packages/release/bioc/html/clusterProfiler.html) to uncover significant pathways from the target genes of key prognosis-associated miRNAs. Statistical analysis was performed using the chi-square test and two-sided Fisher's exact test, with *P* value < 0.05 being considered as statistically significant.

### 2.6. miRNA Gene Network Construction

miRNA gene network was established by the relationship between key miRNAs and their target genes. In the network, genes were denoted by circles, and miRNAs were denoted by rounded rectangles. The network for each miRNA was measured by counting the number of nearby target genes, which were shown as degrees. A higher degree indicated that a miRNA regulated more target genes, implying a more important role in the network.

### 2.7. Real-Time PCR and MACIS Scores

A total of 107 papillary thyroid cancer tissues collected from Shanghai Tenth People's Hospital were used for real-time PCR (qPCR). Total RNA was isolated from tissues using TRIzol reagent (Life Technologies, Carlsbad, CA, USA) and then converted to cDNA using a reverse transcription kit (Takara, Dalian, Liaoning, China). Quantitative PCR was performed in technical triplicates using SYBR Green reagent (Bio-Rad, Hercules, CA, USA). The expression levels were calculated using the 2^–△△Ct^ method, with the Ct values normalized using *β*-actin and U6 as an internal control. The MACIS (Metastases, Age, Completeness of resection, Invasion, Size) prognostic system for PTC was designed at the Mayo Clinic (Rochester, Minnesota, USA) (Supplementary Table S1). This tool has a built-in converter for age stratification (40 years or older), which results in a very accurate correlation between the prognostic score and risk of death. It also disregards node positivity in the calculation of risk of dying, as this is a marker for recurrence rather than disease-specific mortality. Based on evaluation rules, MACIS scores were calculated for 107 papillary thyroid cancer patients, whose detailed information is listed in Supplementary Table S2.

### 2.8. Statistical Analysis

The median, interquartile range, and frequency counts were used to summarize the distribution of clinical data. Fisher's exact test and nonparametric Mann-Whitney *U* test (Wilcoxon rank sum test) were used to test the categorical and continuous variables, respectively. All statistical analyses were conducted with R statistic packages (version 3.6.1; http://www.r-project.org/). The statistical significant level was set at *P* value < 0.05.

## 3. Results

### 3.1. Characteristics of the Patients from the TCGA Papillary Thyroid Cancer Dataset

Based on the data from TCGA, a total of 493 PTC patients with available clinical stage information were studied, including 328 patients in the stage I_II group (stages I and II) and 165 patients in the stage III_IV group (stages III and IV). The clinical features, including gender, age, radiation therapy, neoadjuvant therapy, and survival status, are summarized in Table 1. Although there was no significant difference in gender distribution, patients under 60 years old were more enriched in the stage I_II group (*P* < 0.01). Patients from the stage III_IV group were more likely to receive radiation therapy (*P* < 0.01), whereas only 0.81% of the patients were treated with neoadjuvant therapy, with no significant difference being observed between the two stage groups (*P* = 0.86). Additionally, the patients of stage I and stage II had a significantly higher survival rate than patients of stage III and stage IV (*P* < 0.01).

### 3.2. Differentially Expressed mRNAs and miRNAs between Stage Groups

**Figure 1:**
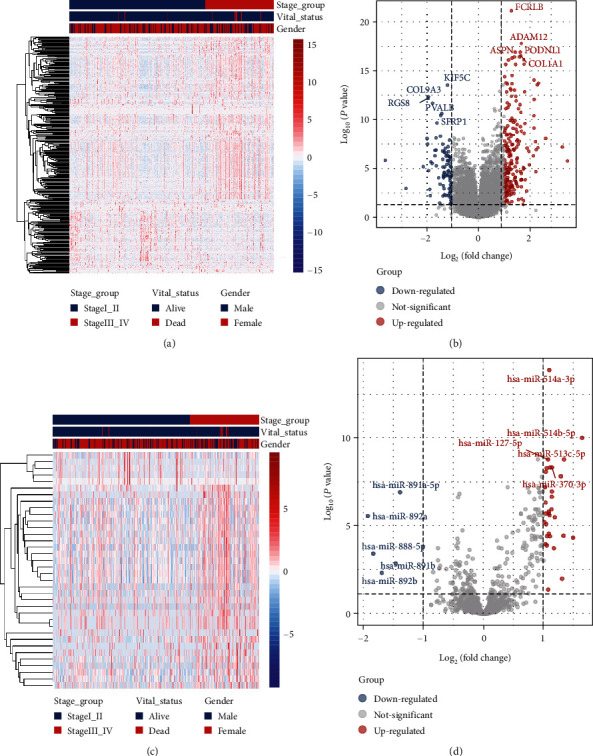
The mRNA and miRNA profiles of different disease stage groups (stage III_IV, *n* = 165; stage I_II, *n* = 328) based on PTC data from TCGA-THCA data collection. (a) Hierarchical clustering of differentially expressed mRNAs in the stage III_IV group (*n* = 165) versus the stage I_II group (*n* = 328). For each gene (row), the red color indicates a higher expression and the blue color indicates a lower expression when compared with the average expression level of that gene across the 493 samples. (b) Volcano plot of mRNA profiles based on the comparison between the stage III_IV group (*n* = 165) and the stage I_II group (*n* = 328). There were 294 differentially expressed genes (filtering criteria: fold change > 2 and *P* < 0.05), of which 202 were upregulated and 92 were downregulated. (c) Hierarchical clustering of differentially expressed miRNAs in the stage III_IV group (*n* = 165) versus the stage I_II group (*n* = 328). For each gene (row), the red color indicates a higher expression and the blue color indicates a lower expression when compared with the average expression level of that gene across the 493 samples. (d) Volcano plot of miRNA profiles based on the comparison between the stage III_IV group (*n* = 165) and the stage I_II group (*n* = 328). There were 36 differentially expressed genes (filtering criteria: fold change > 2 and *P* < 0.05), of which 31 were upregulated and 5 were downregulated.

Hierarchical clustering of differentially expressed mRNAs (Figure 1(a)) and miRNA (Figure 1(c)) was constructed (i.e., supervised clustering based disease stages). The mRNA and miRNA expression profiles were visualized using volcano plots (Figures 1(b) and 1(d)). A total of 294 mRNAs were differentially expressed (Supplementary Table S3), of which 202 (68.71%) were upregulated and 92 (31.29%) were downregulated. On the other hand, 36 miRNAs were differentially expressed between the stage I_II group and the stage III_IV group (Supplementary Table S4), of which 31 (86.11%) were upregulated and 5 (13.89%) were downregulated. The 5 downregulated mRNAs include hsa-miR-891a-5p, hsa-miR-892a, hsa-miR-888-5p, hsa-miR-891b, and hsa-miR-892b (Figure 1(d)).

### 3.3. miRNAs with Significant Prognostic Power

**Figure 2:**
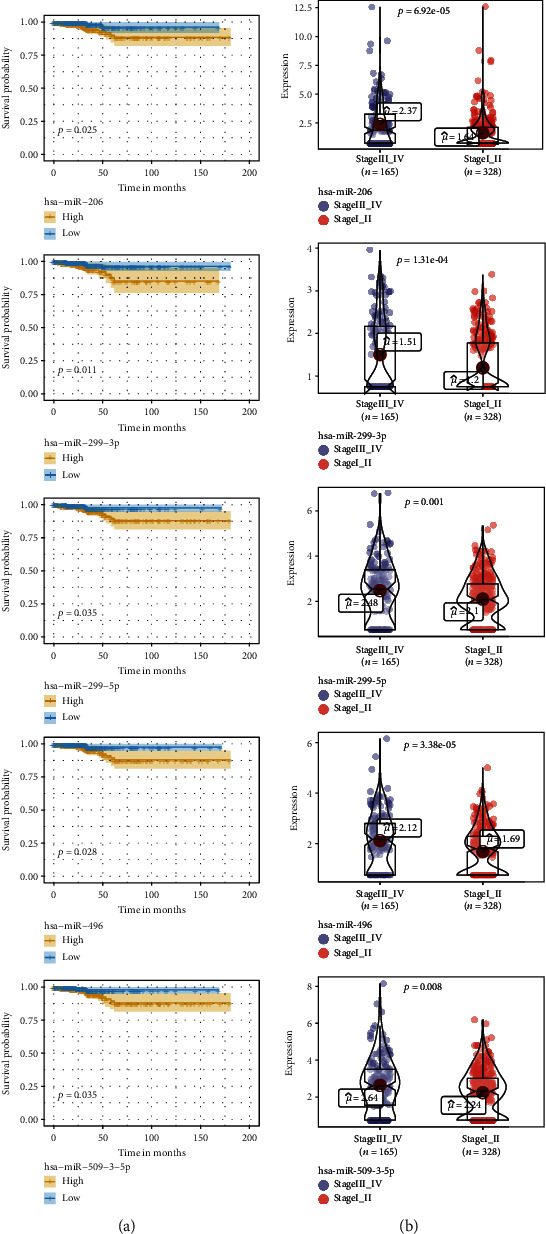
The differentially expressed miRNAs with prognostic implications for all 493 PTC patients from TCGA-THCA data collection. (a) Kaplan-Meier survival analysis of differentially expressed miRNAs (filtering criteria: fold change > 2 and *P* < 0.05), of which 5 miRNAs (hsa-miR-206, hsa-miR-299-3p, hsa-miR-299-5p, hsa-miR-496, and hsa-miR-509-3-5p) were statistically significant (*P* < 0.05). We calculated the hazard ratios (HR) and *P* values with a univariate Cox regression analysis and the log-rank test. OS: overall survival. (b) The expression levels of the five miRNAs (hsa-miR-206, hsa-miR-299-3p, hsa-miR-299-5p, hsa-miR-496, and hsa-miR-509-3-5p) between the 165 stage III_IV patients and the 328 stage I_II patients. *P* values were calculated by the Wilcoxon rank sum test.

The differentially expressed miRNAs with prognostic implications were identified using the Kaplan-Meier survival analysis based on miRNA expression levels in PTC patients. Five signature miRNAs (hsa-miR-206, hsa-miR-299-3p, hsa-miR-299-5p, hsa-miR-496, and hsa-miR-509-3-5p) were found to be significantly associated with the overall survival according to the Cox regression analysis and the log-rank test (*P* < 0.05) (Figure 2(a)). We further analyzed the expression level of the five signature miRNAs between the 165 stage III_IV patients and the 328 stage I_II patients. As illustrated in Figure 2(b), hsa-miR-206 was significantly upregulated in the stage III_IV group versus the stage I_II group (*P* < 0.01). Similar trends were observed for the other four signature miRNAs.

### 3.4. Subgrouping Analysis of the Prognosis-Related miRNAs

**Figure 3:**
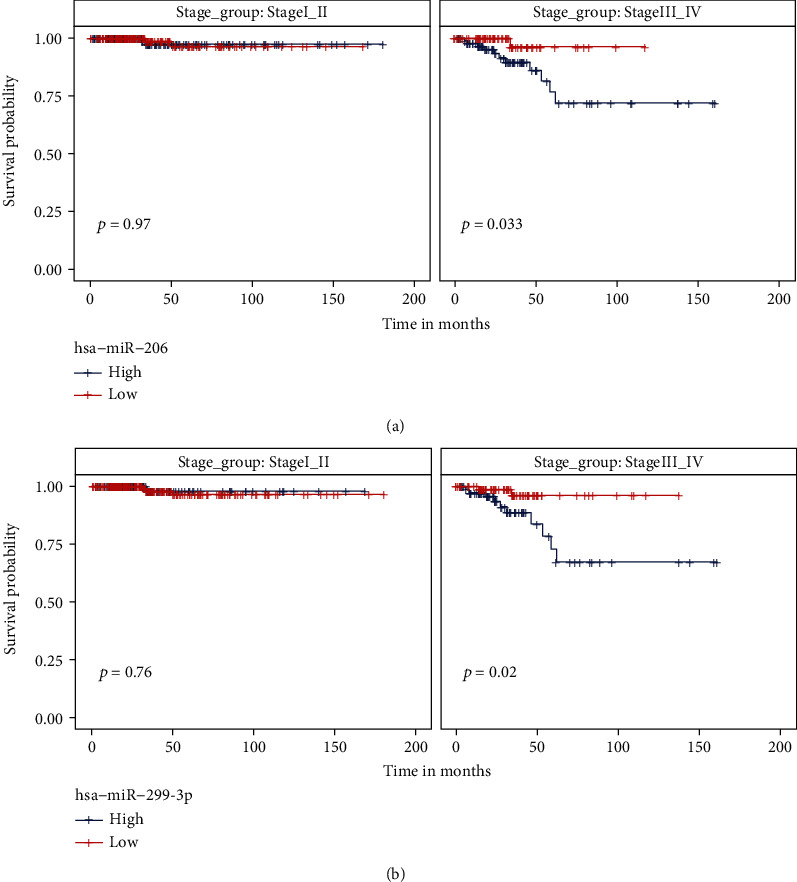
The expression level of hsa-miR-206 and hsa-miR-299-3p was significantly related to prognosis in patients of stage III and stage IV. (a) Kaplan-Meier survival analysis of hsa-miR-206 expression level stratified by stage III_IV and stage I_II patients. (b) Kaplan-Meier survival analysis of hsa-miR-299-3p expression level stratified by stage III_IV and stage I_II patients.

We have identified 5 putative prognosis-related miRNAs that had differential expression levels between stage III_IV and stage I_II patients. In order to rule out the confounding factor of the disease stage during the Kaplan-Meier test, we performed subgrouping analysis to explore the prognostic effect of selected miRNAs in each stage group. As shown in Supplementary Figure S1, the expression level of 3 miRNAs, including hsa-miR-299-5p, hsa-miR-496, and hsa-miR-509-3-5p, failed to significantly separate good and poor prognostic patients within both stage I_II and stage III_IV groups. In contrast, for hsa-miR-206 or hsa-miR-299-3p, patients with low miRNA expression were associated with an improved survival rate than those with high miRNA expression in the stage III-IV group, but not in the stage I_II group (Figure 3).

### 3.5. Predictive Investigation of hsa-miR-206 and hsa-miR-299-3p Functions

**Figure 4:**
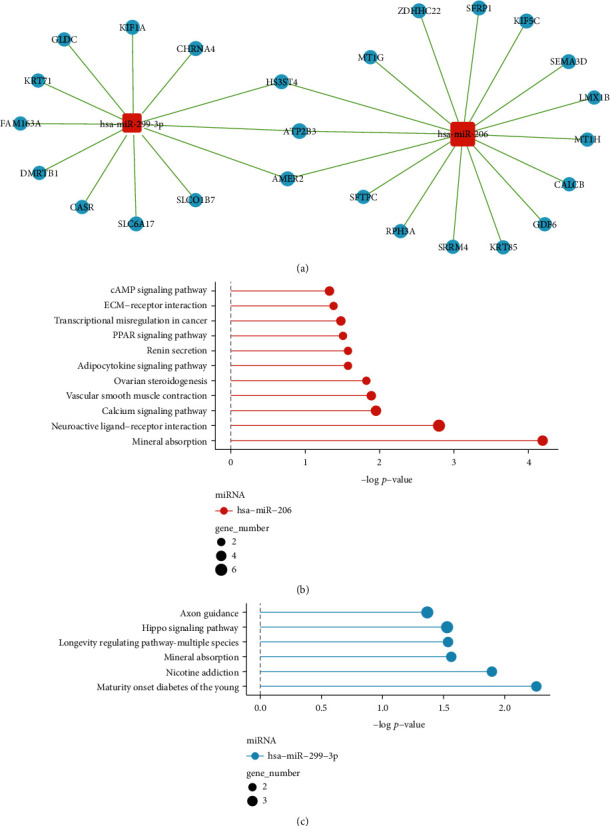
Target prediction and pathway enrichment analysis of hsa-miR-206 and hsa-miR-299-3p. (a) The miRNA-target gene network. In the network, cyan nodes represented miRNAs, and red nodes represented target mRNAs. Edges described the inhibitive effect of miRNAs on mRNAs. (b) Pathway analysis of hsa-miR-206-targeted genes, with only significant pathways being illustrated. The *y*-axis denoted the pathway category and the *x*-axis denoted the –log *P* value) of the pathways. (c) Pathway analysis of hsa-miR-299-3p-targeted genes, with only significant pathways being illustrated. The *y*-axis denoted the pathway category and the *x*-axis denoted the –log *P* value of the pathways.

As the expression level of hsa-miR-206 and hsa-miR-299-3p was associated with both the disease stage in all PTC patients and the prognosis in stage III_IV PTC patients, we estimated the gene targets of these two miRNAs using miRanda and TargetScan databases (see the Materials and Methods selection for more details). The predicted genes were further combined with the differentially expressed mRNA data (Supplementary Table S3) to identify pathologically important target genes of hsa-miR-206 and hsa-miR-299-3p in PTC. Except for a few overlapping genes, such as *HS3ST4*, *ATP2B3*, and *AMER2*, the two miRNAs exhibited relatively distinct miRNA-gene networks (Figure 4(a)). By performing the pathway enrichment analysis, we found that the target genes of hsa-miR-206 were more likely to enrich some oncogenic pathways, including cAMP signaling pathway, extracellular matrix (ECM)-receptor interaction related pathways, and peroxisome proliferator-activated receptor (PPAR) signaling pathway (Figure 4(b) vs.  Figure 4(c)). We, therefore, focused on hsa-miR-206 for the subsequent analysis.

### 3.6. hsa-miR-206 Negatively Regulated the Expression of LMX1B

**Figure 5:**
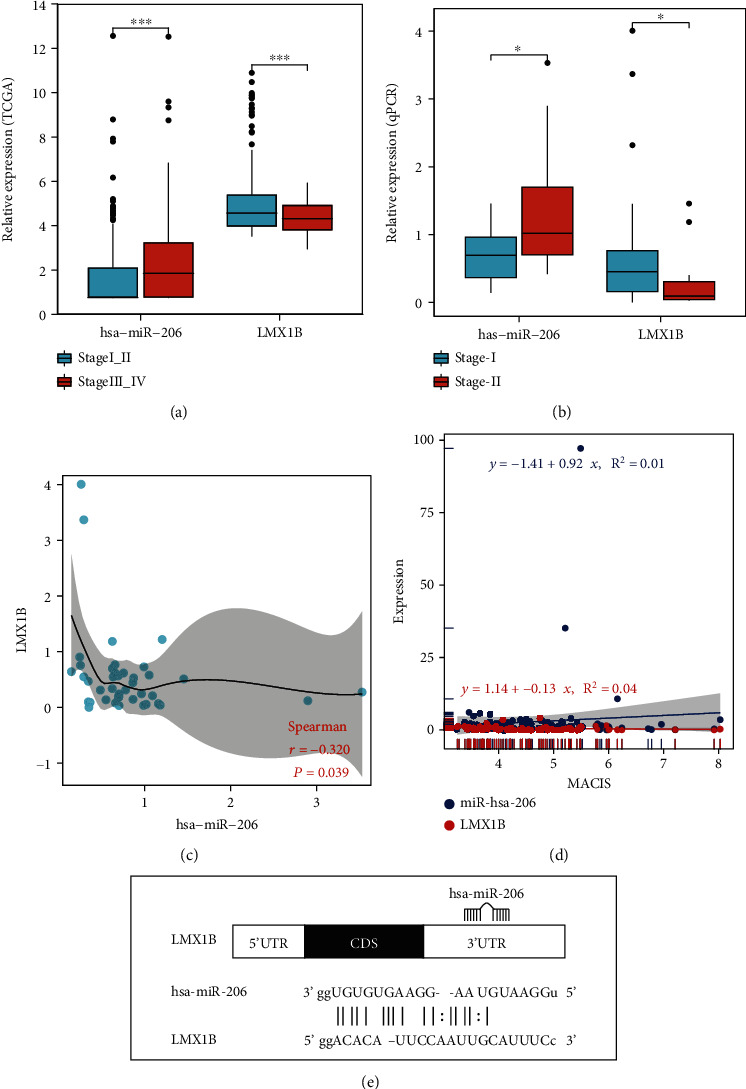
*LMX1B* was a target gene of hsa-miR-206. (a) The *LMX1B* expression level was decreased and the hsa-miR-206 expression level was increased in the stage III_IV group (*n* = 165) compared with the stage I_II group (*n* = 328) based on TCGA data. Boxes represented the interquartile range of the data, and the lines across the boxes indicated the median values. The hash marks above and below the boxes indicated the 90^th^ and 10^th^ percentiles for each group, respectively. *P* values were calculated by the Wilcoxon rank sum test. (b) The *LMX1B* expression level and hsa-miR-206 expression level were analyzed in the stage I and stage II patients based on qPCR data. Boxes represented the interquartile range of the data, and the lines across the boxes indicated the median values. The hash marks above and below the boxes indicated the 90^th^ and 10^th^ percentiles for each group, respectively. *P* values were calculated by the Wilcoxon rank sum test. (c) A significant inverse correlation was observed between the *LMX1B* and hsa-miR-206 expression levels in the PTC tissue samples (*n* = 42) by Pearson's correlation (*P* = 0.039) based on qPCR data. (d) Based on line regression, a negative correlation between the *LMX1B* expression levels and MACIS scores and a positive correlation between the hsa-miR-206 expression levels and MACIS scores were observed. (e) Predicted interaction between hsa-miR-206 and the putative binding sites in the *LMX1B* 3′-UTR based on miRanda. hsa-miR-206 seed sequence was shown in bold. The representation was limited to the region around the hsa-miR-206 complementary site. The number of the binding site was 1959-1981.

We ranked the predicted target gene of hsa-miR-206 based on the log2 fold change of the expression level between the stage III_IV group and the stage I_II group (Supplementary Table S5), as well as the predicted binding affinity between hsa-miR-206 and gene targets (Supplementary Table S6). A few target genes, including *LMX1B*, *MT1H*, *SFRP1*, *SRRM4*, and *SFTPC*, were among the top 10 genes from the two lists and were not shared by the target genes of hsa-miR-299-3p. Additionally, *LMX1B* was the only transcription factor among all the target genes of hsa-miR-206, according to the human TFDB database (Supplementary Table S7). As transcription factors are crucial targets of miRNAs, we further investigated the correlation between *LMX1B* and hsa-miR-206. As shown in Figure 5(a), compared with the expression of hsa-miR-206, *LMX1B* showed an opposite trend of expression between the stage I_II and stage III_IV groups, with the expression level of *LMX1B* being significantly decreased in stage III_IV patients (*P* < 0.01). To further evaluate the expression of *LMX1B* on PTC, qPCR data of stage I and stage II patients were analyzed. The *LMX1B* level was decreased in stage II patients (*n* = 12) compared with stage I patients (*n* = 62) (Figure 5(b)). In contrast, the hsa-miR-206 level was increased in stage II patients (*n* = 11) compared with stage I patients (*n* = 41) (Figure 5(b)). Also, a significant inverse correlation was observed between the *LMX1B* and hsa-miR-206 expression levels in PTC tissues (*n* = 42) by Pearson's correlation (*P* = 0.039) (Figure 5(c)). As shown in Figure 5(d), a negative correlation between the *LMX1B* expression levels and MACIS (distant Metastasis, patient Age, Completeness of resection, local Invasion, and tumor Size) scores and a positive correlation between the hsa-miR-206 expression levels and MACIS scores were detected, indicating that the expressions of *LMX1B* and hsa-miR-206 were likely to correlate with good and bad prognosis in PTC patients, respectively. In order to reveal the interaction between the *LMX1B* and hsa-miR-206, the putative binding sites (site number: 1959-1981) in the *LMX1B* 3′-UTR were predicted (Figure 5(e)). Besides *LMX1B*, the expression and predicted binding sites of other gene targets of hsa-miR-206 are shown in Supplementary Figure S2 and Supplementary Figure S3, respectively.

## 4. Discussion

PTC is a multifactorial disease and has been extensively studied in recent years [6–10]. miRNAs function as either oncogenes or tumor suppressors in PTC tumorigenesis and development [1–5]. With the development of high-throughput sequencing technology, miRNA expression profiles could be established [8, 14, 15]. In this study, mRNA and miRNA sequencing data were achieved from TCGA and used to generate the differentially expressed mRNA and miRNA profiles. The study identified 294 differentially expressed mRNAs and 36 differentially expressed miRNAs in the stage I_II group versus the stage III_IV group. Our analysis revealed 31 upregulated miRNAs and 5 downregulated miRNAs, including hsa-miR-891a-5p, hsa-miR-892a, hsa-miR-888-5p, hsa-miR-891b, and hsa-miR-892b. Our findings were different from the previous research that reported the top 5 deregulated miRNAs of hsa-miR-146b, hsa-miR-375, hsa-miR-31, hsa-miR-7-2, and hsa-miR-204 when comparing lymph node metastasis negative PTC with lymph node metastasis positive PTC [8]. This might be mainly due to the difference in tumor staging of the two studies. In our study, the patients were staged according to the TNM staging classification (stages I, II, III, and IV), while Mutalib et al. used lymph node metastasis status to stratify PTC patients (N0, N1, N1a, and N1b) [8]. Therefore, our results would provide a new understanding of miRNA in the aspect of diagnosis and prognosis for thyroid cancer.

Based on the results acquired by the Cox regression analysis and the log-rank test, five signature miRNAs (hsa-miR-206, hsa-miR-299-3p, hsa-miR-299-5p, hsa-miR-496, and hsa-miR-509-3-5p) were identified as associated with overall survival. All five differentially expressed miRNAs were significantly upregulated in the stage III_IV group versus the stage I_II group. Some of these survival-related miRNAs, including hsa-miR-206 [16, 17] and hsa-miR-299-3p [18], have been studied in thyroid cancer. Chen et al. demonstrated that miR-299-3p acted as a tumor suppressor by regulating *SHOC2* and was usually downregulated in thyroid cancer [18]. Wang et al. showed that hsa-miR-206 inhibited the proliferation and invasion of thyroid cancer by targeting the *RAP1B* gene and hsa-miR-206 negatively regulated the *RAP1B* level in PTC cells [16, 17]. Additionally, the inhibitory effects of hsa-miR-206 on tumor cells were also reported in renal cancer [19], breast cancer [20], colon cancer [21], and ovarian cancer [22]. However, in our study, hsa-miR-206 level was increased in the stage III_IV group compared with the stage I_II group based on TCGA data. The hsa-miR-206 expression levels were also higher in stage II tumors than that in stage I tumors based on qPCR data. This difference might be attributed to the fact that previous studies were based on cancer cells/tissues or normal cells/tissues rather than stratifying patients' tumors by TNM staging. Therefore, our findings provide new insights on the clinical application of hsa-miR-206 in PTC.

The genes targeted by hsa-miR-206 were mostly unknown, and to date, there was only one gene that has been reported as the direct target of this miRNA in PTC. Specifically, Wang et al. reported that *RAP1B*, a member of the small GTPase Ras family, was the target of hsa-miR-206 in PTC. The expression of *RAP1B* was inversely correlated with hsa-miR-206 expression [16, 17]. The negative correlation between miRNA and its target gene was also found in our study. A significant inverse correlation was observed between the *LMX1B* and hsa-miR-206 expression levels in PTC tissues by Pearson's correlation (*P* = 0.039). LMX1B belongs to the LIM-homeodomain- (LIM-HD-) containing family of transcription factors, which play an important role in the development of the midbrain. He et al. reported that cancer cell migration was noticeably promoted by overexpressing *LMX1B* and the migration of human OVCA cells was obviously inhibited when *LMX1B* was knockdown [23]. In our study, *LMX1B* level was significantly decreased in the stage III_IV group (*P* < 0.01) compared with the stage I_II patients based on TCGA data. The *LMX1B* expression level was also lower in stage II patients than that in stage I patients based on qPCR data. These findings revealed a novel mechanism of *LMX1B* as a potential tumor suppressor during PTC disease progression.

In summary, we identified five differentially expressed miRNAs related to thyroid cancer progression and prognosis. We discovered that hsa-miR-206b might be involved in promoting TNM staging in PTC via targeting of *LMX1B*, which is a tumor suppressor in PTC. Future studies with functional experiments are needed to determine the specific functional roles and relationship of the hsa-miR-206b and LMX1B in PTC.

## Figures and Tables

**Table 1:** Clinical characteristics of 493 PTC patients from TCGA-THCA database, stratified by disease stages (stage I_II vs. stage III_IV).

Characteristics	Total	Stage I_II	Stage III_IV	*P* value
No.	493	328	165	
Gender (%)				
Female	360 (73.02)	249 (75.91)	111 (67.27)	0.0533
Male	133 (26.98)	79 (24.09)	54 (32.73)	
Age (%)				
<60	382 (77.48)	284 (86.59)	98 (59.39)	<0.0001^∗∗^
>60	111 (22.52)	44 (13.41)	67 (40.61)	
Radiation therapy (%)				
No	178 (36.10)	143 (43.60)	35 (21.21)	<0.0001^∗∗^
Yes	315 (63.89)	185 (56.40)	130 (78.79)	
Neoadjuvant therapy (%)				
No	489 (99.19)	326 (99.39)	163 (98.79)	0.8638
Yes	4 (0.81)	2 (0.61)	2 (1.21)	
Survival status (%)				
Alive	477 (96.75)	324 (98.78)	153 (92.73)	0.0009^∗∗^
Dead	16 (3.25)	4 (1.22)	12 (7.27)	

Patients of stage I and stage II were categorized as the stage I_II group, and patients of stage III and IV were categorized as the stage III_IV group. *P* value is derived from the univariate association analyses between each of the clinicopathologic variables and stage status. ^∗^*P* value < 0.05 and ^∗∗^*P* value < 0.001.

## Data Availability

All data for this study are presented in the manuscript.

## Supplementary Materials

Supplementary 1 Figure S1: the subgroup analysis of the prognostic effect of the expression level of hsa-miR-299-5p, hsa-miR-496, and hsa-miR-509-3-5p stratified by the disease stage. (A) Kaplan-Meier survival analysis of hsa-miR-299-5p expression level stratified by stage III_IV and stage I_II patients. (B) Kaplan-Meier survival analysis of hsa-miR-496 expression level stratified by stage III_IV and stage I_II patients. (C) Kaplan-Meier survival analysis of hsa-miR-509-3-5p expression level stratified by stage III_IV and stage I_II patients. Figure S2: the expression of target genes of hsa-miR-206 between stage I_II and stage III_IV groups. The differentially expressed genes were analyzed by Deseq2 based on 165 patients of stage III and stage IV and 328 patients of stage I and stage II from TCGA database. Figure S3: the predicted interaction between hsa-miR-206 and its putative target genes based on miRanda.

Supplementary 2 Table S1: the MACIS (Metastases, Age, Completeness of resection, Invasion, and Size) prognostic scoring table for papillary thyroid cancer. Table S2: the detailed clinical characteristics of the 107 papillary thyroid cancer patients. Table S3: the list of differentially expressed mRNA by comparing the stage III_IV group with the stage I_II group. Table S4: the list of differentially expressed miRNA by comparing the stage III_IV group with the stage I_II group. Table S5: the 16 putative target genes of hsa-miR-206 ranked according to the log2 fold change of the expression level between the stage III_IV group and the stage I_II group. Table S6: the 16 putative target genes of hsa-miR-206 ranked according to the binding energy between hsa-miR-206 and the targets. Table S7: the list of transcription factors obtained from the Human TFDB database.
