# Supplementary material for: hsa-miR-206b Involves in the Development of Papillary Thyroid Carcinoma via Targeting LMX1B
Source: Biomed Res Int. 2022 Mar 15;2022:7488708. doi: 10.1155/2022/7488708 (PMC8948606; doi:10.1155/2022/7488708)
Supplement: Supplementary 1 — Figure S1: the subgroup analysis of the prognostic effect of the expression level of hsa-miR-299-5p, hsa-miR-496, and hsa-miR-509-3-5p stratified by the disease stage. (A) Kaplan-Meier survival analysis of hsa-miR-299-5p expression level stratified by stage III_IV and stage I_II patients. (B) Kaplan-Meier survival analysis of hsa-miR-496 expression level stratified by stage III_IV and stage I_II patients. (C) Kaplan-Meier survival analysis of hsa-miR-509-3-5p expression level stratified by stage III_IV and stage I_II patients. Figure S2: the expression of target genes of hsa-miR-206 between stage I_II and stage III_IV groups. The differentially expressed genes were analyzed by Deseq2 based on 165 patients of stage III and stage IV and 328 patients of stage I and stage II from TCGA database. Figure S3: the predicted interaction between hsa-miR-206 and its putative target genes based on miRanda. [file 7488708.f1.zip › Supplementary Figure S3.pdf]

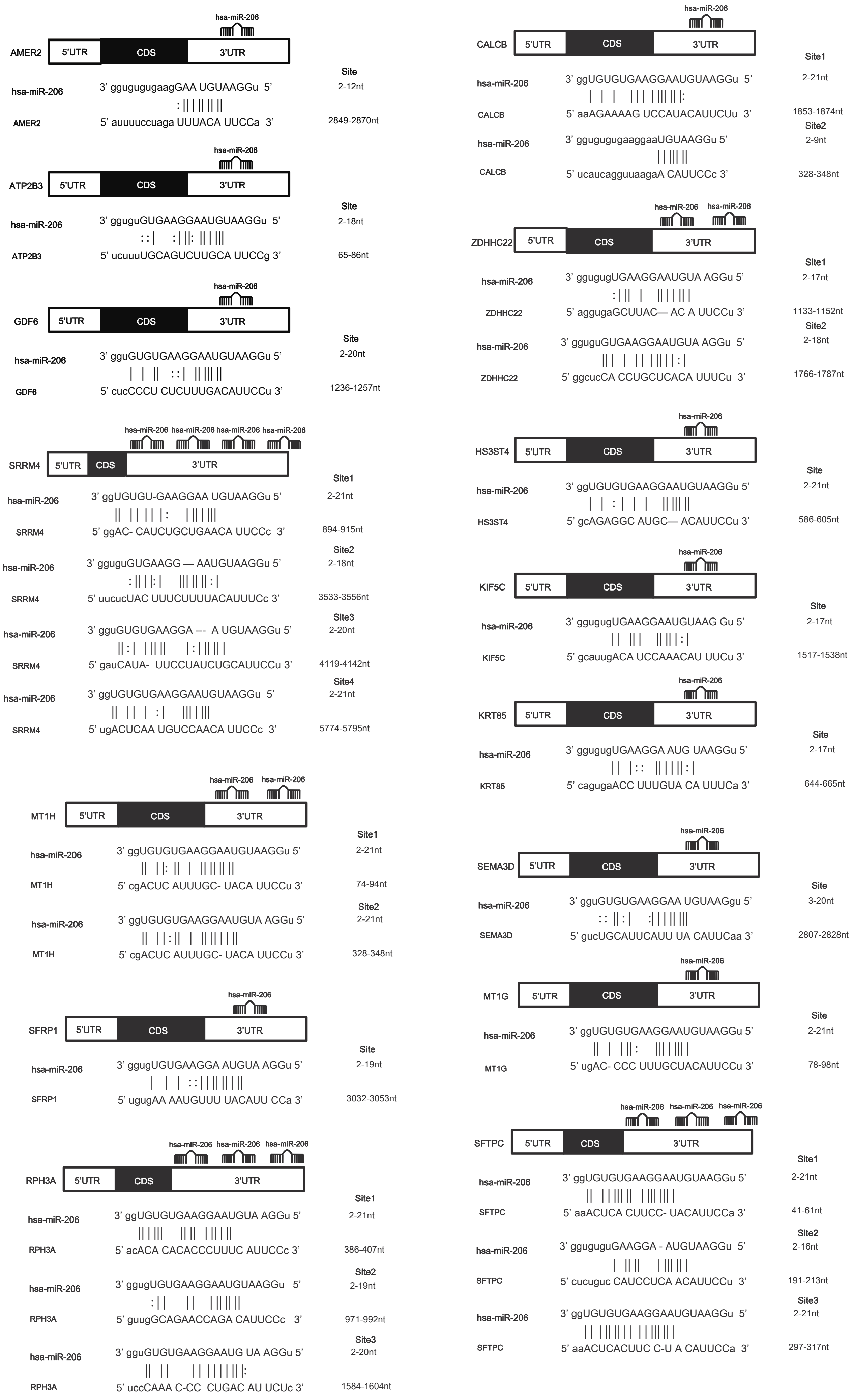

hsa-miR-206
AMER2
5'UTR
CDS
3'UTR
Site
hsa-miR-206
3' ggugugugaagGAA UGUAAGGu 5'
AMER2
5' auuuuccuaga UUUACA UUCCa 3'
2-12nt
2849-2870nt
ATP2B3
Site
hsa-miR-206
3' gguguGUGAAGGAAUGUAAGGu 5'
ATP2B3
5' ucuuuUGCAGUCUUGCA UUCCg 3'
2-18nt
65-86nt
GDF6
Site
hsa-miR-206
3' gguGUGUGAAGGAAUGUAAGGu 5'
GDF6
5' cucCCCU CUCUUUGACAUUCCu 3'
2-20nt
1236-1257nt
SRRM4
Site1
hsa-miR-206
3' ggUGUGU-GAAGGAA UGUAAGGu 5'
SRRM4
5' ggAC- CAUCUGCUGAACA UUCCc 3'
2-21nt
894-915nt
Site2
hsa-miR-206
3' gguguGUGAAGG — AAUGUAAGGu 5'
SRRM4
5' uucucUAC UUUCUUUUACAUUUCc 3'
2-18nt
3533-3556nt
Site3
hsa-miR-206
3' gguGUGUGAAGGA --- A UGUAAGGu 5'
SRRM4
5' gauCAUA- UUCCUAUCUGCAUUCCu 3'
2-20nt
4119-4142nt
Site4
hsa-miR-206
3' ggUGUGUGAAGGAAUGUAAGGu 5'
SRRM4
5' ugACUCAA UGUCCAACA UUCCc 3'
2-21nt
5774-5795nt
MT1H
Site1
hsa-miR-206
3' ggUGUGUGAAGGAAUGUAAGGu 5'
MT1H
5' cgACUC AUUUGC- UACA UUCCu 3'
2-21nt
74-94nt
Site2
hsa-miR-206
3' ggUGUGUGAAGGAAUGUA AGGu 5'
MT1H
5' cgACUC AUUUGC- UACA UUCCu 3'
2-21nt
328-348nt
SFRP1
Site
hsa-miR-206
3' ggugUGUGAAGGA AUGUA AGGu 5'
SFRP1
5' ugugAA AAUGUUU UACAUU CCa 3'
2-19nt
3032-3053nt
RPH3A
Site1
hsa-miR-206
3' ggUGUGUGAAGGAAUGUA AGGu 5'
RPH3A
5' acACA CACACCCUUUC AUUCCc 3'
2-21nt
386-407nt
Site2
hsa-miR-206
3' ggugUGUGAAGGAAUGUAAGGu 5'
RPH3A
5' guugGCAGAACCAGA CAUUCCc 3'
2-19nt
971-992nt
Site3
hsa-miR-206
3' gguGUGUGAAGGAAUG UA AGGu 5'
RPH3A
5' uccCAAA C-CC CUGAC AU UCUc 3'
2-20nt
1584-1604nt
CALCB
Site1
hsa-miR-206
3' ggUGUGUGAAGGAAUGUAAGGu 5'
CALCB
5' aaAGAAAAG UCCAUACAUUCUu 3'
2-21nt
1853-1874nt
Site2
hsa-miR-206
3' ggugugugaaggaaUGUAAGGu 5'
CALCB
5' ucaucagguuaagaA CAUUCCc 3'
2-9nt
328-348nt
ZDHHC22
Site1
hsa-miR-206
3' gguguUGAAGGAAUGUA AGGu 5'
ZDHHC22
5' aggugaGCUUAC— AC A UUCCu 3'
2-17nt
1133-1152nt
Site2
hsa-miR-206
3' gguguGUGAAGGAAUGUA AGGu 5'
ZDHHC22
5' ggcucCA CCUGCUCACA UUUCu 3'
2-18nt
1766-1787nt
HS3ST4
Site
hsa-miR-206
3' ggUGUGUGAAGGAAUGUAAGGu 5'
HS3ST4
5' gcAGAGGC AUGC— ACAUUCCu 3'
2-21nt
586-605nt
KIF5C
Site
hsa-miR-206
3' ggugugUGAAGGAAUGUAAG Gu 5'
KIF5C
5' gcauugACA UCCAAACAU UUCu 3'
2-17nt
1517-1538nt
KRT85
Site
hsa-miR-206
3' ggugugUGAAGGA AUG UAAGGu 5'
KRT85
5' cagugaACC UUUGUA CA UUUCa 3'
2-17nt
644-665nt
SEMA3D
Site
hsa-miR-206
3' gguGUGUGAAGGAA UGUAAGgu 5'
SEMA3D
5' gucUGCAUUCAUU UA CAUUCaa 3'
3-20nt
2807-2828nt
MT1G
Site
hsa-miR-206
3' ggUGUGUGAAGGAAUGUAAGGu 5'
MT1G
5' ugAC- CCC UUUGCUACAUUCCu 3'
2-21nt
78-98nt
SFTPC
Site1
hsa-miR-206
3' ggUGUGUGAAGGAAUGUAAGGu 5'
SFTPC
5' aaACUCA CUUCC- UACAUUCCa 3'
2-21nt
41-61nt
Site2
hsa-miR-206
3' gguguguGAAGGA - AUGUAAGGu 5'
SFTPC
5' cucuguc CAUCCUCA ACAUUCCu 3'
2-16nt
191-213nt
Site3
hsa-miR-206
3' ggUGUGUGAAGGAAUGUAAGGu 5'
SFTPC
5' aaACUCACUUC C-U A CAUUCCa 3'
2-21nt
297-317nt
