# Supplementary material for: hsa-miR-206b Involves in the Development of Papillary Thyroid Carcinoma via Targeting LMX1B
Source: Biomed Res Int. 2022 Mar 15;2022:7488708. doi: 10.1155/2022/7488708 (PMC8948606; doi:10.1155/2022/7488708)
Supplement: Supplementary 1 — Figure S1: the subgroup analysis of the prognostic effect of the expression level of hsa-miR-299-5p, hsa-miR-496, and hsa-miR-509-3-5p stratified by the disease stage. (A) Kaplan-Meier survival analysis of hsa-miR-299-5p expression level stratified by stage III_IV and stage I_II patients. (B) Kaplan-Meier survival analysis of hsa-miR-496 expression level stratified by stage III_IV and stage I_II patients. (C) Kaplan-Meier survival analysis of hsa-miR-509-3-5p expression level stratified by stage III_IV and stage I_II patients. Figure S2: the expression of target genes of hsa-miR-206 between stage I_II and stage III_IV groups. The differentially expressed genes were analyzed by Deseq2 based on 165 patients of stage III and stage IV and 328 patients of stage I and stage II from TCGA database. Figure S3: the predicted interaction between hsa-miR-206 and its putative target genes based on miRanda. [file 7488708.f1.zip › Supplementary figures.docx]

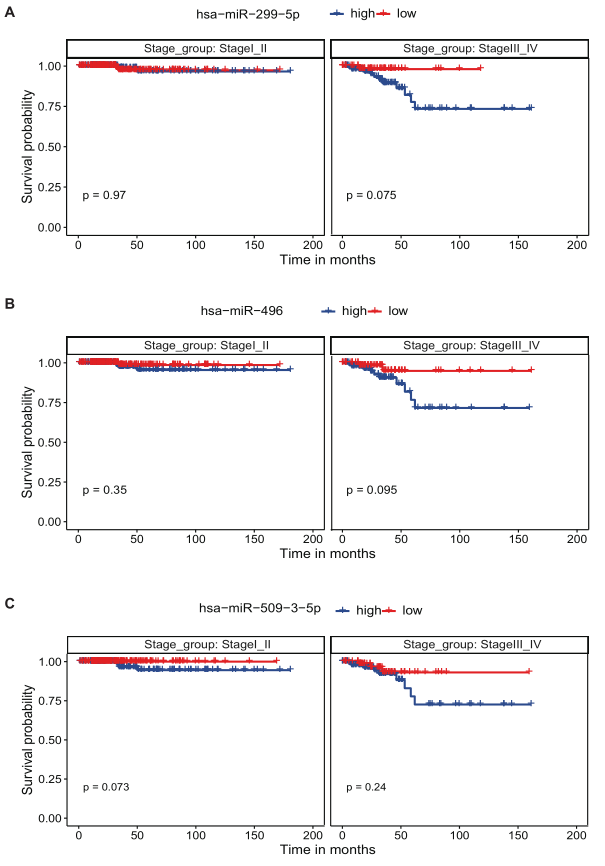


**FIGURE S1:** The subgroup analysis of the prognostic effect of the expression level of hsa-miR-299-5p, hsa-miR-496, and hsa-miR-509-3-5p stratified by the disease stage. (**A**) Kaplan-Meier survival analysis of hsa-miR-299-5p expression level stratified by stage III_IV and stage I_II patients. (**B**) Kaplan-Meier survival analysis of hsa-miR-496 expression level stratified by stage III_IV and stage I_II patients. (**C**) Kaplan-Meier survival analysis of hsa-miR-509-3-5p expression level stratified by stage III_IV and stage I_II patients.

**FIGURE S2:** The expression of target genes of hsa-miR-206 between stage I_II and stage III_IV groups. The differentially expressed genes were analyzed by Deseq2 based on 165 patients of stage III and stage IV and 328 patients of stage I and stage II from TCGA database.

**FIGURE S3:** The predicted interaction between hsa-miR-206 and its putative target genes based on miRanda.
