# Supplementary material for: hsa-miR-206b Involves in the Development of Papillary Thyroid Carcinoma via Targeting LMX1B
Source: Biomed Res Int. 2022 Mar 15;2022:7488708. doi: 10.1155/2022/7488708 (PMC8948606; doi:10.1155/2022/7488708)
Supplement: Supplementary 2 — Table S1: the MACIS (Metastases, Age, Completeness of resection, Invasion, and Size) prognostic scoring table for papillary thyroid cancer. Table S2: the detailed clinical characteristics of the 107 papillary thyroid cancer patients. Table S3: the list of differentially expressed mRNA by comparing the stage III_IV group with the stage I_II group. Table S4: the list of differentially expressed miRNA by comparing the stage III_IV group with the stage I_II group. Table S5: the 16 putative target genes of hsa-miR-206 ranked according to the log2 fold change of the expression level between the stage III_IV group and the stage I_II group. Table S6: the 16 putative target genes of hsa-miR-206 ranked according to the binding energy between hsa-miR-206 and the targets. Table S7: the list of transcription factors obtained from the Human TFDB database. [file 7488708.f2.docx]

**Table S1** The MACIS (Metastases, Age, Completeness of resection, Invasion, Size) prognostic scoring table for papillary thyroid cancer.

| **Characteristics** | **Score** |
| --- | --- |
| **Distant metastasis (spread of cancer to areas outside neck)** |  |
| Yes | 3 |
| No | 0 |
| **Age when tumour discovered (years)** |  |
| ≤39 | 3·1 |
| ≥40 | Age × 0·08 |
| **Invasion into surrounding areas of neck as seen by naked eye** |  |
| Yes | 1 |
| No | 0 |
| **Complete resection (or removal) of tumour** |  |
| No | 1 |
| Yes | 0 |
| **Size of tumour (cm)** | Size × 0·3 |

**Table S2** The detailed clinical characteristics of the 107 papillary thyroid cancer patients.

| **Patient ID** | **Sex** | **Age** | **TMN** | **Pathology** | **Disease stage** | **MACIS score** |
| --- | --- | --- | --- | --- | --- | --- |
| P1 | Female | 43 | T1N1aM0 | Papillary thyroid carcinoma | Ⅰ | 3.89 |
| P2 | Male | 44 | T1N1aM0 | Papillary thyroid carcinoma | Ⅰ | 4.12 |
| P3 | Female | 46 | T1N1aM0 | Papillary thyroid carcinoma | Ⅰ | 4.22 |
| P4 | Female | 21 | T1N1bM0 | Papillary thyroid carcinoma | Ⅰ | 3.46 |
| P5 | Female | 18 | T1N1aM0 | Papillary thyroid carcinoma | Ⅰ | 3.7 |
| P6 | Male | 43 | T1N1aM0 | Papillary thyroid carcinoma | Ⅰ | 3.92 |
| P7 | Female | 28 | T1N1aM0 | Papillary thyroid carcinoma | Ⅰ | 3.7 |
| P8 | Female | 47 | T1N1aM0 | Papillary thyroid carcinoma | Ⅰ | 4.21 |
| P9 | Female | 61 | T2N0M0 | Papillary thyroid carcinoma | Ⅰ | 5.84 |
| P10 | Male | 31 | T1N0M0 | Papillary thyroid carcinoma | Ⅰ | 3.7 |
| P11 | Female | 61 | T1N0M0 | Papillary thyroid carcinoma | Ⅰ | 5.42 |
| P12 | Female | 35 | T1N1bM0 | Papillary thyroid carcinoma | Ⅰ | 3.64 |
| P13 | Male | 32 | T1N1aM0 | Papillary thyroid carcinoma | Ⅰ | 3.55 |
| P14 | Male | 39 | T1N1bM0 | Papillary thyroid carcinoma | Ⅰ | 3.7 |
| P15 | Female | 39 | T1N1bM0 | Papillary thyroid carcinoma | Ⅰ | 3.49 |
| P16 | Female | 67 | T1N1bM0 | Papillary thyroid carcinoma | II | 5.96 |
| P17 | Female | 41 | T1N1bM0 | Papillary thyroid carcinoma | Ⅰ | 3.73 |
| P18 | Female | 22 | T1N1aM0 | Papillary thyroid carcinoma | Ⅰ | 3.55 |
| P19 | Female | 37 | T2N1aM0 | Papillary thyroid carcinoma | Ⅰ | 3.79 |
| P20 | Male | 21 | T2N1aM0 | Papillary thyroid carcinoma | Ⅰ | 3.85 |
| P21 | Male | 38 | T2N1bM0 | Papillary thyroid carcinoma | Ⅰ | 4.15 |
| P22 | Female | 63 | T1N1aM0 | Papillary thyroid carcinoma | II | 5.49 |
| P23 | Female | 34 | T1N1bM0 | Papillary thyroid carcinoma | Ⅰ | 3.55 |
| P24 | Female | 53 | T2N0M0 | Papillary thyroid carcinoma | Ⅰ | 5.14 |
| P25 | Female | 33 | T1N1aM0 | Papillary thyroid carcinoma | Ⅰ | 3.43 |
| P26 | Male | 59 | T1N1aM0 | Papillary thyroid carcinoma | II | 5.32 |
| P27 | Female | 54 | T2N1bM0 | Papillary thyroid carcinoma | Ⅰ | 5.07 |
| P28 | Male | 35 | T2N1bM0 | Papillary thyroid carcinoma | Ⅰ | 3.79 |
| P29 | Female | 55 | T1N1bM0 | Papillary thyroid carcinoma | II | 5 |
| P30 | Female | 40 | T1N0M0 | Papillary thyroid carcinoma | Ⅰ | 3.8 |
| P31 | Female | 67 | T1N1bM0 | Papillary thyroid carcinoma | II | 5.87 |
| P32 | Female | 31 | T4aN1aM0 | Papillary thyroid carcinoma | Ⅰ | 3.85 |
| P33 | Female | 36 | T2N1aM0 | Papillary thyroid carcinoma | Ⅰ | 3.85 |
| P34 | Male | 72 | T3N0M0 | Papillary thyroid carcinoma | II | 6.96 |
| P35 | Female | 22 | T2N1bM0 | Papillary thyroid carcinoma | Ⅰ | 4 |
| P36 | Male | 61 | T1N1aM0 | Papillary thyroid carcinoma | II | 5.21 |
| P37 | Male | 39 | T1N1aM0 | Papillary thyroid carcinoma | Ⅰ | 3.61 |
| P38 | Male | 48 | T1N0M0 | Papillary thyroid carcinoma | Ⅰ | 4.08 |
| P39 | Female | 57 | T1N1aM0 | Papillary thyroid carcinoma | II | 6.16 |
| P40 | Male | 57 | T1N1aM0 | Papillary thyroid carcinoma | II | 4.56 |
| P41 | Female | 52 | T4aN1aM0 | Papillary thyroid carcinoma | I | 6 |
| P42 | Female | 58 | T1N1aM0 | Papillary thyroid carcinoma | II | 6.24 |
| P43 | Male | 41 | T4aN1aM0 | Papillary thyroid carcinoma | I | 4.73 |
| P44 | Male | 46 | T1N1bM0 | Papillary thyroid carcinoma | I | 4.98 |
| P45 | Male | 26 | T1N1aM0 | Papillary thyroid carcinoma | I | 4.55 |
| P46 | Female | 46 | T1N1aM0 | Papillary thyroid carcinoma | I | 4.13 |
| P47 | Female | 64 | T1N1aM0 | Papillary thyroid carcinoma | II | 6.72 |
| P48 | Female | 49 | T1N1aM0 | Papillary thyroid carcinoma | I | 5.31 |
| P49 | Female | 44 | T2N1aM0 | Papillary thyroid carcinoma | I | 5.48 |
| P50 | Female | 79 | T1N0M0 | Papillary thyroid carcinoma | I | 7.92 |
| P51 | Female | 38 | T1N1aM0 | Papillary thyroid carcinoma | I | 3.43 |
| P52 | Female | 45 | T1N0M0 | Papillary thyroid carcinoma | I | 4.14 |
| P53 | Female | 46 | T1N0M0 | Papillary thyroid carcinoma | I | 4.04 |
| P54 | Female | 30 | T1N0M0 | Papillary thyroid carcinoma | I | 3.58 |
| P55 | Male | 30 | T1N0M0 | Papillary thyroid carcinoma | I | 3.385 |
| P56 | Male | 42 | T1N1aM0 | Papillary thyroid carcinoma | I | 3.81 |
| P57 | Female | 39 | T1N1aM0 | Papillary thyroid carcinoma | I | 3.46 |
| P58 | Female | 33 | T1N1aM0 | Papillary thyroid carcinoma | I | 3.46 |
| P59 | Female | 32 | T4aN0M0 | Papillary thyroid carcinoma | I | 3.55 |
| P60 | Male | 47 | T1N1aM0 | Papillary thyroid carcinoma | I | 4.21 |
| P61 | Female | 56 | T1N1aM0 | Papillary thyroid carcinoma | I | 4.78 |
| P62 | Female | 55 | T1N0M0 | Papillary thyroid carcinoma | I | 4.88 |
| P63 | Male | 52 | T1N1aM0 | Papillary thyroid carcinoma | I | 4.61 |
| P64 | Female | 48 | T1N1aM0 | Papillary thyroid carcinoma | I | 4.08 |
| P65 | Female | 59 | T1N0M0 | Papillary thyroid carcinoma | I | 4.75 |
| P66 | Female | 68 | T1N1aM0 | Papillary thyroid carcinoma | I | 5.8 |
| P67 | Male | 55 | T1N1aM0 | Papillary thyroid carcinoma | I | 4.58 |
| P68 | Female | 44 | T1N0M0 | Papillary thyroid carcinoma | I | 3.79 |
| P69 | Male | 62 | T1N0M0 | Papillary thyroid carcinoma | I | 5.29 |
| P70 | Female | 49 | T1N0M0 | Papillary thyroid carcinoma | I | 4.25 |
| P71 | Female | 36 | T1N0M0 | Papillary thyroid carcinoma | I | 3.28 |
| P72 | Female | 34 | T1N1aM0 | Papillary thyroid carcinoma | I | 3.55 |
| P73 | Female | 49 | T1N1aM0 | Papillary thyroid carcinoma | I | 4.28 |
| P74 | Male | 47 | T1N1aM0 | Papillary thyroid carcinoma | I | 5.27 |
| P75 | Female | 27 | T1N1aM0 | Papillary thyroid carcinoma | I | 4.43 |
| P76 | Female | 31 | T1N1aM0 | Papillary thyroid carcinoma | I | 4.43 |
| P77 | Female | 46 | T1N1aM0 | Papillary thyroid carcinoma | I | 3.83 |
| P78 | Female | 25 | T1N1aM0 | Papillary thyroid carcinoma | I | 3.25 |
| P79 | Female | 42 | T1N1aM0 | Papillary thyroid carcinoma | I | 3.663 |
| P80 | Female | 57 | T1N0M0 | Papillary thyroid carcinoma | I | 4.95 |
| P81 | Female | 54 | T2N1aM0 | Papillary thyroid carcinoma | I | 5.07 |
| P82 | Female | 60 | T1N1aM0 | Papillary thyroid carcinoma | I | 5.16 |
| P83 | Female | 61 | T1N1aM0 | Papillary thyroid carcinoma | II | 5.48 |
| P84 | Male | 68 | T1N0M0 | Papillary thyroid carcinoma | I | 5.77 |
| P85 | Female | 52 | T1N1aM0 | Papillary thyroid carcinoma | I | 5.49 |
| P86 | Female | 29 | T1N1aM0 | Papillary thyroid carcinoma | I | 4.55 |
| P87 | Female | 45 | T1N1aM0 | Papillary thyroid carcinoma | I | 3.96 |
| P88 | Female | 51 | T1N1aM0 | Papillary thyroid carcinoma | I | 4.53 |
| P89 | Female | 41 | T1N1aM0 | Papillary thyroid carcinoma | I | 3.73 |
| P90 | Female | 44 | T1N0M0 | Papillary thyroid carcinoma | I | 3.88 |
| P91 | Female | 48 | T2N1aM0 | Papillary thyroid carcinoma | I | 4.59 |
| P92 | Female | 56 | T1N1aM0 | Papillary thyroid carcinoma | I | 4.81 |
| P93 | Male | 51 | T1N0M0 | Papillary thyroid carcinoma | I | 4.395 |
| P94 | Female | 29 | T2N1aM0 | Papillary thyroid carcinoma | I | 4 |
| P95 | Male | 57 | T1bN1aM0 | Papillary thyroid carcinoma | II | 6.01 |
| P96 | Female | 56 | T1bN1aM0 | Papillary thyroid carcinoma | II | 5.99 |
| P97 | Female | 46 | T1bN0M0 | Papillary thyroid carcinoma | I | 4.19 |
| P98 | Female | 48 | T1bN0M0 | Papillary thyroid carcinoma | I | 4.2 |
| P99 | Male | 51 | T1bN1aM0 | Papillary thyroid carcinoma | I | 4.5 |
| P100 | Male | 30 | T2N1bM0 | Papillary thyroid carcinoma | I | 4.85 |
| P101 | Female | 78 | T1bN1aM0 | Papillary thyroid carcinoma | II | 6.78 |
| P102 | Female | 39 | T1bN1aM0 | Papillary thyroid carcinoma | I | 4.49 |
| P103 | Female | 36 | T1bN1aM0 | Papillary thyroid carcinoma | I | 4.43 |
| P104 | Male | 54 | T1bN1aM0 | Papillary thyroid carcinoma | I | 4.92 |
| P105 | Male | 44 | T1bN1bM1 | Papillary thyroid carcinoma | II | 8.03 |
| P106 | Female | 72 | T3bN0M0 | Papillary thyroid carcinoma | II | 7.21 |
| P107 | Female | 49 | T1bN0M0 | Papillary thyroid carcinoma | I | 5.52 |

**Table S3** The list of differentially expressed mRNA by comparing the stage III_IV group with the stage I_II group.

| **Ensembl ID** | **Symbol** | **Biotype** | **log2 Fold Change** | ***P*-value** | **Trend** |
| --- | --- | --- | --- | --- | --- |
| ENSG00000148848 | ADAM12 | protein_coding | 1.663593349 | 1.64E-18 | up |
| ENSG00000138316 | ADAMTS14 | protein_coding | 1.098020861 | 8.38E-08 | up |
| ENSG00000181092 | ADIPOQ | protein_coding | 1.657674569 | 0.00074131 | up |
| ENSG00000188778 | ADRB3 | protein_coding | 1.025401516 | 9.43E-06 | up |
| ENSG00000111732 | AICDA | protein_coding | -1.083994673 | 0.002295793 | down |
| ENSG00000198074 | AKR1B10 | protein_coding | 1.157247467 | 3.15E-05 | up |
| ENSG00000165566 | AMER2 | protein_coding | -1.295869621 | 6.65E-05 | down |
| ENSG00000230062 | ANKRD66 | protein_coding | -1.0657623 | 0.002548369 | down |
| ENSG00000104537 | ANXA13 | protein_coding | 1.0620592 | 0.000288018 | up |
| ENSG00000198768 | APCDD1L | protein_coding | 1.184233534 | 6.48E-07 | up |
| ENSG00000110244 | APOA4 | protein_coding | -1.36573589 | 0.006417048 | down |
| ENSG00000062096 | ARSF | protein_coding | -1.124024281 | 0.003290685 | down |
| ENSG00000183876 | ARSI | protein_coding | 1.41270528 | 1.12E-10 | up |
| ENSG00000106819 | ASPN | protein_coding | 1.416207373 | 1.22E-17 | up |
| ENSG00000067842 | ATP2B3 | protein_coding | -1.781887773 | 3.15E-06 | down |
| ENSG00000172232 | AZU1 | protein_coding | 1.036325371 | 1.72E-05 | up |
| ENSG00000125888 | BANF2 | protein_coding | -1.33395596 | 2.80E-05 | down |
| ENSG00000101197 | BIRC7 | protein_coding | 1.372520343 | 1.44E-07 | up |
| ENSG00000204347 | BTBD17 | protein_coding | -1.624318148 | 5.54E-06 | down |
| ENSG00000183346 | C10orf107 | protein_coding | -1.080694536 | 7.89E-07 | down |
| ENSG00000150750 | C11orf53 | protein_coding | 1.43353269 | 8.07E-05 | up |
| ENSG00000082196 | C1QTNF3 | protein_coding | 1.015527315 | 3.92E-15 | up |
| ENSG00000180044 | C3orf80 | protein_coding | 1.05218693 | 2.21E-16 | up |
| ENSG00000138813 | C4orf17 | protein_coding | 1.022115363 | 0.000835411 | up |
| ENSG00000110680 | CALCA | protein_coding | -1.347232831 | 9.02E-05 | down |
| ENSG00000175868 | CALCB | protein_coding | -1.594971059 | 1.97E-10 | down |
| ENSG00000203697 | CAPN8 | protein_coding | 1.079810795 | 4.02E-09 | up |
| ENSG00000164326 | CARTPT | protein_coding | -1.921720429 | 1.70E-05 | down |
| ENSG00000036828 | CASR | protein_coding | -1.956687519 | 3.52E-08 | down |
| ENSG00000163394 | CCKAR | protein_coding | 1.514376104 | 0.000684441 | up |
| ENSG00000172156 | CCL11 | protein_coding | 1.175053753 | 0.001601713 | up |
| ENSG00000102970 | CCL17 | protein_coding | 1.49299877 | 2.80E-10 | up |
| ENSG00000102962 | CCL22 | protein_coding | 1.108322583 | 1.68E-07 | up |
| ENSG00000204936 | CD177 | protein_coding | 1.078664784 | 0.002783802 | up |
| ENSG00000158477 | CD1A | protein_coding | 1.0431646 | 0.000281211 | up |
| ENSG00000071991 | CDH19 | protein_coding | 1.416501189 | 6.74E-06 | up |
| ENSG00000147889 | CDKN2A | protein_coding | 1.136389853 | 5.09E-12 | up |
| ENSG00000124469 | CEACAM8 | protein_coding | 1.171657129 | 0.000488733 | up |
| ENSG00000189052 | CGB5 | protein_coding | 1.675179307 | 0.012205066 | up |
| ENSG00000070748 | CHAT | protein_coding | 1.982626158 | 4.00E-06 | up |
| ENSG00000101204 | CHRNA4 | protein_coding | -1.150967034 | 1.72E-05 | down |
| ENSG00000157884 | CIB4 | protein_coding | -1.101030878 | 0.000107635 | down |
| ENSG00000187288 | CIDEC | protein_coding | 1.106254822 | 3.03E-05 | up |
| ENSG00000137975 | CLCA2 | protein_coding | 1.213458768 | 3.09E-06 | up |
| ENSG00000018236 | CNTN1 | protein_coding | 1.188356583 | 6.53E-06 | up |
| ENSG00000123500 | COL10A1 | protein_coding | 1.648711741 | 1.60E-11 | up |
| ENSG00000060718 | COL11A1 | protein_coding | 2.334268939 | 1.92E-14 | up |
| ENSG00000111799 | COL12A1 | protein_coding | 1.301320299 | 4.95E-17 | up |
| ENSG00000108821 | COL1A1 | protein_coding | 1.639880094 | 2.89E-17 | up |
| ENSG00000164692 | COL1A2 | protein_coding | 1.385047624 | 3.82E-17 | up |
| ENSG00000168542 | COL3A1 | protein_coding | 1.437762202 | 2.21E-16 | up |
| ENSG00000130635 | COL5A1 | protein_coding | 1.299388917 | 2.98E-15 | up |
| ENSG00000204262 | COL5A2 | protein_coding | 1.066545562 | 1.65E-14 | up |
| ENSG00000163359 | COL6A3 | protein_coding | 1.111105723 | 1.75E-12 | up |
| ENSG00000092758 | COL9A3 | protein_coding | -1.926164894 | 4.19E-13 | down |
| ENSG00000145244 | CORIN | protein_coding | 1.059333808 | 6.72E-12 | up |
| ENSG00000178772 | CPN2 | protein_coding | 1.218794462 | 1.77E-07 | up |
| ENSG00000169509 | CRCT1 | protein_coding | 2.240154051 | 1.18E-07 | up |
| ENSG00000198930 | CSAG1 | protein_coding | -1.374547284 | 1.23E-06 | down |
| ENSG00000129170 | CSRP3 | protein_coding | 2.226847131 | 1.96E-05 | up |
| ENSG00000164932 | CTHRC1 | protein_coding | 1.391877916 | 8.72E-14 | up |
| ENSG00000169862 | CTNND2 | protein_coding | -1.009627918 | 2.79E-05 | down |
| ENSG00000111249 | CUX2 | protein_coding | -1.673321677 | 5.43E-07 | down |
| ENSG00000163739 | CXCL1 | protein_coding | 1.124905148 | 6.57E-08 | up |
| ENSG00000163735 | CXCL5 | protein_coding | 1.389767047 | 1.09E-07 | up |
| ENSG00000095596 | CYP26A1 | protein_coding | 1.76263455 | 7.37E-17 | up |
| ENSG00000174325 | DIRC1 | protein_coding | 1.748432924 | 7.54E-06 | up |
| ENSG00000143006 | DMRTB1 | protein_coding | -1.831501883 | 2.24E-06 | down |
| ENSG00000169676 | DRD5 | protein_coding | 1.204646428 | 0.000235411 | up |
| ENSG00000102385 | DRP2 | protein_coding | 1.321480261 | 3.76E-08 | up |
| ENSG00000197561 | ELANE | protein_coding | 1.123822555 | 0.000151535 | up |
| ENSG00000083782 | EPYC | protein_coding | 2.130953509 | 4.93E-07 | up |
| ENSG00000143340 | FAM163A | protein_coding | -1.186356715 | 4.96E-07 | down |
| ENSG00000204767 | FAM196B | protein_coding | -1.047711104 | 0.000776743 | down |
| ENSG00000188100 | FAM25A | protein_coding | 1.823407448 | 0.000137398 | up |
| ENSG00000187773 | FAM69C | protein_coding | 1.168073012 | 9.51E-09 | up |
| ENSG00000147689 | FAM83A | protein_coding | 1.06479881 | 3.15E-06 | up |
| ENSG00000078098 | FAP | protein_coding | 1.297618681 | 1.98E-14 | up |
| ENSG00000165323 | FAT3 | protein_coding | 1.291446796 | 6.31E-11 | up |
| ENSG00000077942 | FBLN1 | protein_coding | 1.002899073 | 4.88E-08 | up |
| ENSG00000162746 | FCRLB | protein_coding | 1.271179582 | 7.69E-22 | up |
| ENSG00000214814 | FER1L6 | protein_coding | -1.079348665 | 0.000466959 | down |
| ENSG00000171560 | FGA | protein_coding | 1.727845643 | 0.04484079 | up |
| ENSG00000171564 | FGB | protein_coding | 1.87981784 | 0.001889113 | up |
| ENSG00000162344 | FGF19 | protein_coding | 1.263553706 | 0.002206767 | up |
| ENSG00000105550 | FGF21 | protein_coding | -1.87495191 | 0.00134231 | down |
| ENSG00000138675 | FGF5 | protein_coding | 1.171611475 | 0.003137711 | up |
| ENSG00000126500 | FLRT1 | protein_coding | -1.014235205 | 2.92E-05 | down |
| ENSG00000172461 | FUT9 | protein_coding | 1.41304163 | 8.37E-05 | up |
| ENSG00000136542 | GALNT5 | protein_coding | 1.296249189 | 1.15E-10 | up |
| ENSG00000215644 | GCGR | protein_coding | -1.294082881 | 7.09E-06 | down |
| ENSG00000156466 | GDF6 | protein_coding | -1.12585325 | 3.07E-06 | down |
| ENSG00000121743 | GJA3 | protein_coding | -1.115715213 | 1.38E-08 | down |
| ENSG00000178445 | GLDC | protein_coding | -1.316270808 | 2.35E-07 | down |
| ENSG00000214415 | GNAT3 | protein_coding | -2.125788386 | 6.74E-06 | down |
| ENSG00000115523 | GNLY | protein_coding | 1.127308721 | 3.60E-09 | up |
| ENSG00000257008 | GPR142 | protein_coding | -1.180116476 | 4.19E-05 | down |
| ENSG00000173612 | GPRC6A | protein_coding | 1.457140627 | 0.000327001 | up |
| ENSG00000134443 | GRP | protein_coding | 1.058481072 | 0.000434665 | up |
| ENSG00000048545 | GUCA1A | protein_coding | 1.26460978 | 1.26E-05 | up |
| ENSG00000105509 | HAS1 | protein_coding | 1.571874444 | 1.79E-08 | up |
| ENSG00000180818 | HOXC10 | protein_coding | 1.275377261 | 3.23E-06 | up |
| ENSG00000037965 | HOXC8 | protein_coding | 1.079120944 | 2.91E-08 | up |
| ENSG00000182601 | HS3ST4 | protein_coding | -1.033684274 | 0.000288111 | down |
| ENSG00000029559 | IBSP | protein_coding | 1.060461753 | 3.50E-05 | up |
| ENSG00000188293 | IGFL1 | protein_coding | 1.478785253 | 1.24E-06 | up |
| ENSG00000188624 | IGFL3 | protein_coding | 2.154431988 | 4.02E-11 | up |
| ENSG00000128322 | IGLL1 | protein_coding | -1.07832408 | 6.47E-05 | down |
| ENSG00000254709 | IGLL5 | protein_coding | -1.128607518 | 0.001148304 | down |
| ENSG00000095752 | IL11 | protein_coding | 1.046205665 | 5.02E-08 | up |
| ENSG00000138684 | IL21 | protein_coding | -1.050422965 | 0.044587065 | down |
| ENSG00000164485 | IL22RA2 | protein_coding | 1.081749344 | 0.000586028 | up |
| ENSG00000136694 | IL36A | protein_coding | 1.081990805 | 0.002623093 | up |
| ENSG00000136688 | IL36G | protein_coding | 1.232233787 | 0.000808592 | up |
| ENSG00000125571 | IL37 | protein_coding | 2.15740072 | 7.22E-10 | up |
| ENSG00000168348 | INSM2 | protein_coding | 1.49072075 | 1.60E-09 | up |
| ENSG00000027644 | INSRR | protein_coding | 1.544292008 | 6.81E-12 | up |
| ENSG00000159387 | IRX6 | protein_coding | -1.249954905 | 4.40E-05 | down |
| ENSG00000016082 | ISL1 | protein_coding | -1.097825064 | 0.008584323 | down |
| ENSG00000198542 | ITGBL1 | protein_coding | 1.13007306 | 3.89E-10 | up |
| ENSG00000163207 | IVL | protein_coding | 1.814298251 | 2.47E-10 | up |
| ENSG00000177301 | KCNA2 | protein_coding | -1.030640384 | 5.52E-05 | down |
| ENSG00000162989 | KCNJ3 | protein_coding | -1.159989295 | 2.00E-05 | down |
| ENSG00000157542 | KCNJ6 | protein_coding | 1.453173758 | 8.35E-10 | up |
| ENSG00000139330 | KERA | protein_coding | 1.960439367 | 1.64E-07 | up |
| ENSG00000196169 | KIF19 | protein_coding | -1.449981428 | 3.29E-11 | down |
| ENSG00000130294 | KIF1A | protein_coding | -1.376986719 | 3.56E-08 | down |
| ENSG00000168280 | KIF5C | protein_coding | -1.192672558 | 2.75E-14 | down |
| ENSG00000174562 | KLK15 | protein_coding | -1.041087018 | 0.000774708 | down |
| ENSG00000129455 | KLK8 | protein_coding | 1.288549114 | 3.40E-06 | up |
| ENSG00000131737 | KRT34 | protein_coding | 1.103426958 | 0.01716573 | up |
| ENSG00000205420 | KRT6A | protein_coding | 1.485676663 | 2.65E-06 | up |
| ENSG00000170465 | KRT6C | protein_coding | 1.062617787 | 0.022570099 | up |
| ENSG00000139648 | KRT71 | protein_coding | -1.634980541 | 8.05E-09 | down |
| ENSG00000170454 | KRT75 | protein_coding | 1.74009629 | 1.15E-05 | up |
| ENSG00000185640 | KRT79 | protein_coding | 1.000657449 | 4.11E-05 | up |
| ENSG00000135443 | KRT85 | protein_coding | -1.673356654 | 1.46E-07 | down |
| ENSG00000196734 | LCE1B | protein_coding | 1.065657685 | 0.00208978 | up |
| ENSG00000197084 | LCE1C | protein_coding | 1.218740281 | 0.00077408 | up |
| ENSG00000160349 | LCN1 | protein_coding | 1.04285864 | 0.000157935 | up |
| ENSG00000136110 | LECT1 | protein_coding | -1.20531062 | 5.75E-05 | down |
| ENSG00000143768 | LEFTY2 | protein_coding | -1.199862585 | 1.61E-06 | down |
| ENSG00000204021 | LIPK | protein_coding | 1.368694699 | 0.003514 | up |
| ENSG00000170807 | LMOD2 | protein_coding | 1.259345102 | 0.000218346 | up |
| ENSG00000136944 | LMX1B | protein_coding | -1.21556074 | 3.87E-06 | down |
| ENSG00000113083 | LOX | protein_coding | 1.051190934 | 6.63E-10 | up |
| ENSG00000117598 | LPPR5 | protein_coding | 2.213070748 | 1.73E-08 | up |
| ENSG00000172061 | LRRC15 | protein_coding | 2.157608955 | 8.93E-15 | up |
| ENSG00000176204 | LRRTM4 | protein_coding | -1.030017544 | 0.020600021 | down |
| ENSG00000139329 | LUM | protein_coding | 1.259004938 | 3.19E-09 | up |
| ENSG00000132031 | MATN3 | protein_coding | 1.113538681 | 2.14E-14 | up |
| ENSG00000183019 | MCEMP1 | protein_coding | 1.34486632 | 1.23E-07 | up |
| ENSG00000145794 | MEGF10 | protein_coding | 1.216459416 | 2.70E-09 | up |
| ENSG00000203740 | METTL11B | protein_coding | 2.602076137 | 8.24E-09 | up |
| ENSG00000197614 | MFAP5 | protein_coding | 1.604744263 | 2.63E-12 | up |
| ENSG00000096395 | MLN | protein_coding | -1.356465219 | 0.000564486 | down |
| ENSG00000102539 | MLNR | protein_coding | -1.14913553 | 1.61E-06 | down |
| ENSG00000196549 | MME | protein_coding | 1.02720455 | 1.50E-06 | up |
| ENSG00000142606 | MMEL1 | protein_coding | -1.151900509 | 2.04E-07 | down |
| ENSG00000166670 | MMP10 | protein_coding | 1.800052981 | 1.43E-13 | up |
| ENSG00000099953 | MMP11 | protein_coding | 1.096496927 | 2.97E-09 | up |
| ENSG00000137745 | MMP13 | protein_coding | 2.308300133 | 7.04E-08 | up |
| ENSG00000137675 | MMP27 | protein_coding | 1.383350603 | 0.001508661 | up |
| ENSG00000149968 | MMP3 | protein_coding | 1.393301197 | 0.000134177 | up |
| ENSG00000137673 | MMP7 | protein_coding | 1.115700888 | 0.00013847 | up |
| ENSG00000185038 | MROH2A | protein_coding | -1.083917139 | 0.005418459 | down |
| ENSG00000125144 | MT1G | protein_coding | -1.295758957 | 2.63E-05 | down |
| ENSG00000205358 | MT1H | protein_coding | -1.633996881 | 1.40E-07 | down |
| ENSG00000204544 | MUC21 | protein_coding | 1.162170557 | 2.73E-05 | up |
| ENSG00000133055 | MYBPH | protein_coding | 1.177646501 | 1.52E-07 | up |
| ENSG00000109061 | MYH1 | protein_coding | 1.081402157 | 0.005996288 | up |
| ENSG00000125414 | MYH2 | protein_coding | 3.240077576 | 6.09E-08 | up |
| ENSG00000133020 | MYH8 | protein_coding | 1.027703944 | 0.009320661 | up |
| ENSG00000168530 | MYL1 | protein_coding | 3.432796517 | 1.60E-06 | up |
| ENSG00000122180 | MYOG | protein_coding | 1.161725787 | 0.001565354 | up |
| ENSG00000170476 | MZB1 | protein_coding | -1.065875923 | 0.000997999 | down |
| ENSG00000109705 | NKX3-2 | protein_coding | 1.456168141 | 1.08E-12 | up |
| ENSG00000109255 | NMU | protein_coding | 1.159425423 | 2.12E-05 | up |
| ENSG00000163273 | NPPC | protein_coding | -1.051142123 | 2.78E-05 | down |
| ENSG00000122585 | NPY | protein_coding | -1.35699496 | 6.98E-05 | down |
| ENSG00000197893 | NRAP | protein_coding | 2.166177349 | 8.64E-13 | up |
| ENSG00000174145 | NWD2 | protein_coding | -1.533256631 | 8.99E-05 | down |
| ENSG00000144460 | NYAP2 | protein_coding | -1.351896323 | 4.77E-05 | down |
| ENSG00000104044 | OCA2 | protein_coding | -1.051158675 | 7.23E-06 | down |
| ENSG00000106809 | OGN | protein_coding | 1.133075221 | 8.88E-06 | up |
| ENSG00000127083 | OMD | protein_coding | 1.249469508 | 3.81E-07 | up |
| ENSG00000183269 | OR52E8 | protein_coding | 1.543928702 | 0.014539894 | up |
| ENSG00000125813 | PAX1 | protein_coding | -1.028233897 | 0.009204997 | down |
| ENSG00000162366 | PDZK1IP1 | protein_coding | 1.039765008 | 4.85E-06 | up |
| ENSG00000124102 | PI3 | protein_coding | 1.245954107 | 5.43E-07 | up |
| ENSG00000164093 | PITX2 | protein_coding | 1.785904414 | 2.46E-09 | up |
| ENSG00000132000 | PODNL1 | protein_coding | 1.615795846 | 1.22E-17 | up |
| ENSG00000133110 | POSTN | protein_coding | 1.581930465 | 3.82E-17 | up |
| ENSG00000203805 | PPAPDC1A | protein_coding | 1.338643621 | 6.63E-10 | up |
| ENSG00000163736 | PPBP | protein_coding | 1.073379031 | 1.28E-05 | up |
| ENSG00000074211 | PPP2R2C | protein_coding | -1.080969712 | 6.36E-06 | down |
| ENSG00000108849 | PPY | protein_coding | -1.249486406 | 0.005386604 | down |
| ENSG00000204479 | PRAMEF17 | protein_coding | -1.47913902 | 0.000408825 | down |
| ENSG00000224940 | PRRT4 | protein_coding | -1.246751299 | 2.92E-07 | down |
| ENSG00000221826 | PSG3 | protein_coding | 1.293285978 | 0.000645023 | up |
| ENSG00000170848 | PSG6 | protein_coding | 1.44124179 | 0.000210614 | up |
| ENSG00000221878 | PSG7 | protein_coding | 1.313088138 | 0.000894716 | up |
| ENSG00000183668 | PSG9 | protein_coding | 1.230475363 | 0.000849073 | up |
| ENSG00000054356 | PTPRN | protein_coding | 1.358348799 | 1.67E-10 | up |
| ENSG00000100362 | PVALB | protein_coding | -1.768831365 | 1.94E-12 | down |
| ENSG00000184672 | RALYL | protein_coding | -1.046572678 | 0.031804646 | down |
| ENSG00000104918 | RETN | protein_coding | 1.501854053 | 1.30E-08 | up |
| ENSG00000251258 | RFPL4B | protein_coding | 1.288233947 | 0.000485398 | up |
| ENSG00000135824 | RGS8 | protein_coding | -1.943217531 | 6.16E-13 | down |
| ENSG00000104237 | RP1 | protein_coding | 1.025388508 | 7.21E-05 | up |
| ENSG00000259680 | RP11-812E19.9 | protein_coding | -1.308076507 | 0.004769446 | down |
| ENSG00000089169 | RPH3A | protein_coding | -1.167068306 | 6.48E-08 | down |
| ENSG00000196754 | S100A2 | protein_coding | 1.272787282 | 3.78E-13 | up |
| ENSG00000196154 | S100A4 | protein_coding | 1.013967312 | 1.74E-09 | up |
| ENSG00000163220 | S100A9 | protein_coding | 1.028175235 | 1.09E-09 | up |
| ENSG00000173432 | SAA1 | protein_coding | 1.247817354 | 1.16E-05 | up |
| ENSG00000134339 | SAA2 | protein_coding | 1.00104593 | 0.001068977 | up |
| ENSG00000255071 | SAA2-SAA4 | protein_coding | 1.295254155 | 0.000607663 | up |
| ENSG00000148965 | SAA4 | protein_coding | 1.031584464 | 0.003510082 | up |
| ENSG00000153993 | SEMA3D | protein_coding | -1.094651769 | 0.000184041 | down |
| ENSG00000124233 | SEMG1 | protein_coding | -2.792339714 | 0.001078065 | down |
| ENSG00000186910 | SERPINA11 | protein_coding | 1.655919936 | 3.33E-05 | up |
| ENSG00000165953 | SERPINA12 | protein_coding | 1.470534759 | 0.000361888 | up |
| ENSG00000170099 | SERPINA6 | protein_coding | 1.181213414 | 0.032525989 | up |
| ENSG00000206072 | SERPINB11 | protein_coding | -3.595207867 | 1.56E-06 | down |
| ENSG00000197632 | SERPINB2 | protein_coding | 1.129060318 | 8.66E-05 | up |
| ENSG00000206073 | SERPINB4 | protein_coding | 1.096337589 | 0.017739004 | up |
| ENSG00000166396 | SERPINB7 | protein_coding | 1.534519943 | 3.76E-08 | up |
| ENSG00000104332 | SFRP1 | protein_coding | -1.412162438 | 2.13E-11 | down |
| ENSG00000145423 | SFRP2 | protein_coding | 1.478478132 | 7.07E-08 | up |
| ENSG00000106483 | SFRP4 | protein_coding | 1.185457977 | 3.73E-07 | up |
| ENSG00000120057 | SFRP5 | protein_coding | 1.663227927 | 1.35E-09 | up |
| ENSG00000122852 | SFTPA1 | protein_coding | 1.132087382 | 0.003852825 | up |
| ENSG00000185303 | SFTPA2 | protein_coding | 1.501201392 | 8.95E-07 | up |
| ENSG00000168484 | SFTPC | protein_coding | -1.277811746 | 9.34E-08 | down |
| ENSG00000107295 | SH3GL2 | protein_coding | -1.31975605 | 1.91E-07 | down |
| ENSG00000132874 | SLC14A2 | protein_coding | -1.465078535 | 5.49E-09 | down |
| ENSG00000187714 | SLC18A3 | protein_coding | 1.770380789 | 8.78E-06 | up |
| ENSG00000135917 | SLC19A3 | protein_coding | 1.104930215 | 1.92E-07 | up |
| ENSG00000155886 | SLC24A2 | protein_coding | 1.338853754 | 3.94E-05 | up |
| ENSG00000182747 | SLC35D3 | protein_coding | 1.279261584 | 1.61E-06 | up |
| ENSG00000115665 | SLC5A7 | protein_coding | -1.979671882 | 9.51E-09 | down |
| ENSG00000197106 | SLC6A17 | protein_coding | -1.079726882 | 1.35E-06 | down |
| ENSG00000205754 | SLCO1B7 | protein_coding | -1.088861628 | 0.03765292 | down |
| ENSG00000185985 | SLITRK2 | protein_coding | -1.25041685 | 5.93E-07 | down |
| ENSG00000170290 | SLN | protein_coding | 2.280710722 | 2.75E-14 | up |
| ENSG00000124107 | SLPI | protein_coding | 1.260007388 | 5.71E-09 | up |
| ENSG00000126233 | SLURP1 | protein_coding | 1.307826319 | 0.000652092 | up |
| ENSG00000115593 | SMYD1 | protein_coding | 1.287866386 | 0.023734854 | up |
| ENSG00000156395 | SORCS3 | protein_coding | 1.688084837 | 0.000141501 | up |
| ENSG00000167941 | SOST | protein_coding | 1.081905855 | 0.006439459 | up |
| ENSG00000134595 | SOX3 | protein_coding | -1.065875444 | 0.017201843 | down |
| ENSG00000178287 | SPAG11A | protein_coding | -1.494154386 | 0.033941291 | down |
| ENSG00000077327 | SPAG6 | protein_coding | 1.180203088 | 1.25E-05 | up |
| ENSG00000134668 | SPOCD1 | protein_coding | 1.002724194 | 1.64E-08 | up |
| ENSG00000262655 | SPON1 | protein_coding | 1.15617134 | 1.18E-07 | up |
| ENSG00000169474 | SPRR1A | protein_coding | 1.218569936 | 0.005085792 | up |
| ENSG00000169469 | SPRR1B | protein_coding | 1.442833926 | 0.000210881 | up |
| ENSG00000163216 | SPRR2D | protein_coding | 1.658090326 | 0.000137385 | up |
| ENSG00000244094 | SPRR2F | protein_coding | 1.762034126 | 0.018183208 | up |
| ENSG00000163209 | SPRR3 | protein_coding | 1.006691877 | 0.030357769 | up |
| ENSG00000175093 | SPSB4 | protein_coding | 1.184915676 | 1.37E-07 | up |
| ENSG00000277893 | SRD5A2 | protein_coding | 1.218574365 | 1.96E-08 | up |
| ENSG00000102359 | SRPX2 | protein_coding | 1.232859997 | 6.23E-14 | up |
| ENSG00000139767 | SRRM4 | protein_coding | -1.384902609 | 1.75E-07 | down |
| ENSG00000157005 | SST | protein_coding | 1.48816656 | 1.96E-05 | up |
| ENSG00000171772 | SYCE1 | protein_coding | -1.367002804 | 7.46E-06 | down |
| ENSG00000067715 | SYT1 | protein_coding | 1.353267583 | 3.63E-09 | up |
| ENSG00000166220 | TBATA | protein_coding | -1.326262634 | 0.001140907 | down |
| ENSG00000134827 | TCN1 | protein_coding | 1.092363097 | 0.000210614 | up |
| ENSG00000164362 | TERT | protein_coding | 1.641686178 | 0.000154588 | up |
| ENSG00000186340 | THBS2 | protein_coding | 1.193114179 | 2.70E-12 | up |
| ENSG00000258986 | TMEM179 | protein_coding | -1.138753638 | 6.59E-07 | down |
| ENSG00000087128 | TMPRSS11E | protein_coding | 1.045083964 | 0.001047748 | up |
| ENSG00000117586 | TNFSF4 | protein_coding | 1.160700095 | 7.65E-17 | up |
| ENSG00000169344 | UMOD | protein_coding | -1.854921236 | 0.005884039 | down |
| ENSG00000038427 | VCAN | protein_coding | 1.225405997 | 1.08E-10 | up |
| ENSG00000134258 | VTCN1 | protein_coding | 1.077784361 | 0.000803293 | up |
| ENSG00000182931 | WFDC10B | protein_coding | 1.286730338 | 4.08E-08 | up |
| ENSG00000243543 | WFDC6 | protein_coding | -1.165757079 | 0.005689242 | down |
| ENSG00000156076 | WIF1 | protein_coding | 1.270031095 | 0.004263733 | up |
| ENSG00000104415 | WISP1 | protein_coding | 1.442308375 | 3.79E-13 | up |
| ENSG00000064205 | WISP2 | protein_coding | 1.781596993 | 2.21E-16 | up |
| ENSG00000105989 | WNT2 | protein_coding | 1.477325094 | 1.68E-08 | up |
| ENSG00000154764 | WNT7A | protein_coding | 1.166491123 | 2.65E-05 | up |
| ENSG00000184937 | WT1 | protein_coding | 1.622444265 | 4.59E-10 | up |
| ENSG00000158125 | XDH | protein_coding | 1.106263915 | 2.65E-05 | up |
| ENSG00000124343 | XG | protein_coding | 1.253297192 | 8.72E-14 | up |
| ENSG00000179300 | ZCCHC5 | protein_coding | 1.114394064 | 0.000171016 | up |
| ENSG00000177108 | ZDHHC22 | protein_coding | -1.115187899 | 6.59E-07 | down |
| ENSG00000152977 | ZIC1 | protein_coding | 1.893716886 | 7.69E-08 | up |
| ENSG00000174963 | ZIC4 | protein_coding | 1.629413924 | 2.15E-05 | up |
| ENSG00000225614 | ZNF469 | protein_coding | 1.088096857 | 1.35E-13 | up |
| ENSG00000198597 | ZNF536 | protein_coding | -1.105001094 | 7.27E-05 | down |

**Table S4** The list of differentially expressed miRNA by comparing the stage III_IV group with the stage I_II group.

| **MicroRNA ID** | **log2 Fold Change** | ***P* value** | **Trend** |
| --- | --- | --- | --- |
| hsa-miR-122-5p | 1.06971629 | 4.38E-02 | up |
| hsa-miR-127-3p | 1.04555498 | 5.62E-09 | up |
| hsa-miR-127-5p | 1.067913499 | 1.75E-09 | up |
| hsa-miR-134-3p | 1.005788363 | 6.35497E-06 | up |
| hsa-miR-154-5p | 1.016061934 | 6.57E-08 | up |
| hsa-miR-206 | 1.479385337 | 4.89E-05 | up |
| hsa-miR-299-3p | 1.1805557 | 3.45153E-06 | up |
| hsa-miR-299-5p | 1.026374576 | 4.97E-07 | up |
| hsa-miR-323a-3p | 1.078156337 | 2.09E-06 | up |
| hsa-miR-369-5p | 1.031925136 | 2.08E-06 | up |
| hsa-miR-370-3p | 1.131942901 | 5.05E-09 | up |
| hsa-miR-376a-5p | 1.091861805 | 2.64E-06 | up |
| hsa-miR-376b-5p | 1.051439015 | 4.03E-05 | up |
| hsa-miR-376c-5p | 1.07959089 | 2.88E-05 | up |
| hsa-miR-377-5p | 1.276968809 | 1.59E-08 | up |
| hsa-miR-379-3p | 1.023520072 | 1.13E-04 | up |
| hsa-miR-382-3p | 1.131688964 | 1.17E-07 | up |
| hsa-miR-382-5p | 1.031908931 | 9.02E-09 | up |
| hsa-miR-412-5p | 1.037369225 | 8.30147E-06 | up |
| hsa-miR-431-3p | 1.101446069 | 5.05E-09 | up |
| hsa-miR-496 | 1.129756951 | 2.32E-07 | up |
| hsa-miR-509-3-5p | 1.125629574 | 1.21066E-06 | up |
| hsa-miR-509-5p | 1.300306589 | 1.06E-02 | up |
| hsa-miR-513a-5p | 1.164482607 | 1.94E-04 | up |
| hsa-miR-513c-5p | 1.329312576 | 1.75E-09 | up |
| hsa-miR-514a-3p | 1.087948648 | 1.48E-14 | up |
| hsa-miR-514a-5p | 1.079781453 | 1.62E-06 | up |
| hsa-miR-514b-5p | 1.629151211 | 1.039E-10 | up |
| hsa-miR-541-3p | 1.321217091 | 3.76E-05 | up |
| hsa-miR-656-3p | 1.098662016 | 4.02E-05 | up |
| hsa-miR-7156-5p | 1.047068137 | 1.35E-04 | up |
| hsa-miR-888-5p | -1.823355066 | 0.000397515 | down |
| hsa-miR-891a-5p | -1.381671791 | 1.27357E-07 | down |
| hsa-miR-891b | -1.452264273 | 0.001454374 | down |
| hsa-miR-892a | -1.913237141 | 2.92E-06 | down |
| hsa-miR-892b | -1.680487139 | 0.004928696 | down |

**Table S5** The 16 putative target genes of hsa-miR-206 ranked according to the log2 fold change of the expression level between the stage III_IV group and the stage I_II group.

| **Rank** | **Symbol** | **log2 Fold Change** | ***P*-value** |
| --- | --- | --- | --- |
| 1 | ATP2B3 | -1.781887773 | 3.15E-06 |
| 2 | KRT85 | -1.673356654 | 1.46E-07 |
| 3 | MT1H | -1.633996881 | 1.40E-07 |
| 4 | CALCB | -1.594971059 | 1.97E-10 |
| 5 | SFRP1 | -1.412162438 | 2.13E-11 |
| 6 | SRRM4 | -1.384902609 | 1.75E-07 |
| 7 | AMER2 | -1.295869621 | 6.65E-05 |
| 8 | MT1G | -1.295758957 | 2.63E-05 |
| 9 | SFTPC | -1.277811746 | 9.34E-08 |
| 10 | LMX1B | -1.21556074 | 3.87E-06 |
| 11 | KIF5C | -1.19267 | 2.75E-14 |
| 12 | RPH3A | -1.16707 | 6.48E-08 |
| 13 | GDF6 | -1.12585 | 3.07E-06 |
| 14 | ZDHHC22 | -1.11519 | 6.59E-07 |
| 15 | SEMA3D | -1.09465 | 0.000184 |
| 16 | HS3ST4 | -1.03368 | 0.000288 |

**Table S6** The 16 putative target genes of hsa-miR-206 ranked according to the binding energy between hsa-miR-206 and the targets.

| **Rank** | **Gene_Symbol** | **Scores** | **Energy** | **Alignment length** |
| --- | --- | --- | --- | --- |
| 1 | SFTPC | 178 | -27.95 | 19 |
| 2 | RPH3A | 152 | -26.58 | 19 |
| 3 | LMX1B | 141 | -19.33 | 21 |
| 4 | MT1H | 166 | -19.1 | 19 |
| 5 | ATP2B3 | 141 | -18.83 | 16 |
| 6 | SRRM4 | 144 | -18.76 | 21 |
| 7 | AMER2 | 151 | -17.67 | 10 |
| 8 | GDF6 | 155 | -16.05 | 18 |
| 9 | SFRP1 | 158 | -15.71 | 17 |
| 10 | SEMA3D | 143 | -15.06 | 17 |
| 11 | MT1G | 158 | -14.97 | 19 |
| 12 | ZDHHC22 | 145 | -14.78 | 16 |
| 13 | KIF5C | 140 | -14.4 | 15 |
| 14 | KRT85 | 140 | -13.9 | 15 |
| 15 | CALCB | 144 | -12.65 | 19 |
| 16 | HS3ST4 | 141 | -12.64 | 19 |

**Table S7** The list of transcription factors obtained from the Human TFDB database.

| ZBTB8B | HSFX1 | TERF1 | PRDM12 | ZIK1 | AC105001.2 |
| --- | --- | --- | --- | --- | --- |
| GSX2 | FOXC1 | RXRG | TSHZ2 | ZNF611 | TEF |
| TBX2 | PAX9 | USF2 | KLF13 | DLX2 | PPARA |
| PAX8 | ZNF28 | ZNF35 | FOXO6 | RREB1 | ZNF8 |
| CREB3L1 | MNT | FOXO4 | IRX2 | GATA1 | ZNF501 |
| NKX6-1 | NEUROD2 | RFX2 | MYBL1 | BHLHA15 | ZNF454 |
| ZNF621 | ZNF133 | CTCFL | CEBPA | SOX21 | PRDM2 |
| ZNF138 | BNC1 | AFF1 | ZNF407 | MAFA | ETS1 |
| ZNF654 | PRDM9 | ZNF775 | MIXL1 | PITX1 | PRDM6 |
| POU3F3 | HNF1A | ELF2 | MEIS1 | ZNF286B | ZBED3 |
| ALX1 | ZNF653 | ZNF32 | ZNF671 | TGIF2LX | ZNF354C |
| EMX1 | UNCX | LHX5 | AC138696.1 | SATB1 | ESX1 |
| ZNF718 | TGIF2 | SOHLH1 | ZNF630 | SIM2 | HOXB6 |
| VSX2 | ZSCAN32 | NSD2 | ZNF524 | EN1 | NFKB1 |
| POU1F1 | ZFHX4 | HMGB4 | MEF2B | ZNF845 | TULP3 |
| RARA | ZFP69 | ZBTB44 | ZNF662 | DRGX | RARG |
| TLX3 | ETV7 | ZNF444 | ZNF534 | MAX | ZNF705G |
| ZIC2 | TLX1 | NKX2-5 | PRDM5 | ZNF696 | OLIG3 |
| ZNF226 | ZNF747 | ARID5B | TOX2 | ZNF521 | ZNF821 |
| ZNF808 | ATF7 | JDP2 | ZNF415 | ZFP62 | HNF4A |
| AC067968.1 | ZNF74 | FOS | ZBTB20 | GLI3 | TCF7 |
| NR3C1 | ETV3L | BARX1 | ZNF740 | ZBTB8A | SMAD4 |
| HIC2 | FIZ1 | LIN28B | SPIC | ZNF791 | DMRTC2 |
| ZNF442 | ZNF177 | LHX3 | TBX15 | ZFP42 | ZBED5 |
| FOSL1 | ZKSCAN1 | NRF1 | ZNF766 | TGIF1 | NEUROG3 |
| MTF1 | HOXA7 | HOXC13 | ZNF25 | ZNF557 | ESR2 |
| YBX3 | GATA5 | FOXS1 | PMS1 | MEIS3 | ZNF683 |
| ZNF705A | THAP3 | DMTF1 | MYC | ZNF565 | ZEB1 |
| NFE2 | TFEB | ISL1 | SIX6 | AC002310.5 | NPAS3 |
| RAX2 | SOX3 | STAT3 | POU2F3 | DLX1 | ZNF785 |
| SP1 | MYSM1 | ETV6 | EHF | OLIG1 | HOXA4 |
| ARID3A | HEY2 | IRX3 | ZNF544 | GCFC2 | ZNF831 |
| FEZF1 | ZNF84 | ZNF627 | NANOGNB | ZNF124 | HOXB5 |
| THAP4 | NCOA1 | ZKSCAN5 | ZBTB43 | ZBTB5 | HMGXB3 |
| ZNF286A | SUB1 | NFATC4 | ZNF536 | ZNF554 | ZNF227 |
| FOXQ1 | ZNF723 | ZNF212 | ZNF440 | ZBTB38 | ZNF446 |
| AIRE | SOX13 | KLF5 | RORB | ZNF600 | PAXBP1 |
| SNAI3 | ZBTB37 | ZNF572 | ZNF844 | BSX | HSFX2 |
| ZNF224 | MSX1 | HES1 | ZNF263 | BHLHE41 | ARID4B |
| ATOH8 | ZNF564 | ZNF343 | ZNF846 | ZSCAN23 | ZNF157 |
| MECOM | NKX2-1 | ZNF91 | MTA2 | HOXA1 | MXD4 |
| ZNF45 | ZNF292 | THAP7 | CBFB | MLX | LRRFIP1 |
| ZNF674 | ZNF749 | GTF2IRD2B | NPAS1 | TSC22D1 | ZFP30 |
| ZXDA | LHX4 | ONECUT1 | AC022167.5 | EGR4 | ELF3 |
| ST18 | HES2 | ZNF79 | ZNF57 | DUXA | INSM2 |
| NKX6-2 | HLF | ZSCAN5C | EN2 | ZNF493 | HMGA1 |
| ZNF689 | IRF9 | TRERF1 | ZFPM2 | CREBZF | MITF |
| ZNF449 | PPARG | BATF | HESX1 | PAX1 | MXI1 |
| ZNF2 | SHOX2 | LHX9 | SP3 | THAP11 | NR4A1 |
| TFDP2 | ZNF277 | TOX4 | E2F8 | ARID4A | ZNF585B |
| ZBTB25 | ZNF878 | REL | ZNF563 | ZSCAN22 | TBX21 |
| PIAS4 | ARID1B | HOXD11 | MAFK | ZNF729 | TFAP2C |
| HES4 | ZNF426 | ZBTB34 | ZNF569 | ZNF221 | HMG20A |
| ZNF592 | DLX4 | ATF6B | ZNF300 | FOXN1 | ZNF770 |
| WT1 | ZNF33B | FOXD4L6 | HES7 | MSGN1 | KLF6 |
| ZBTB49 | ZNF639 | ELK3 | NR1I2 | OTX2 | GLI2 |
| PAX5 | BARHL2 | ZNF559 | SOX7 | TCF7L1 | SIX4 |
| MAFG | ZNF83 | GSC2 | ZBTB39 | THAP6 | ZNF813 |
| ZNF837 | HAND2 | ONECUT3 | ZNF398 | IRF2 | ZNF7 |
| FOXD4 | ZBTB42 | ARNT | ZNF420 | TFAP2D | FOXK2 |
| GATA3 | ARNTL2 | ETV4 | NCOR1 | ZNF239 | ZNF577 |
| HOMEZ | ZNF320 | ZNF792 | SHOX | EBF1 | RHOXF2B |
| ZNF26 | ZNF10 | IRF1 | CEBPE | GBX1 | ZNF623 |
| ZSCAN20 | TUB | NFATC3 | LITAF | REST | USF3 |
| MEOX2 | ZNF107 | PAX2 | SEBOX | CSDE1 | SOX12 |
| DMRTA1 | ZFP36L1 | ZNF222 | ZBTB11 | ZNF641 | HSF4 |
| ZNF567 | ZNF713 | KLF4 | FOXP2 | ZNF90 | ZNF705E |
| OVOL1 | ARID2 | TSC22D3 | TLX2 | FOXN3 | GCM2 |
| PRRX2 | ATF4 | ZNF229 | TOX3 | ZNF385A | E2F4 |
| MAFB | KMT2C | TFEC | NR3C2 | ZNF727 | FOXP1 |
| ZNF195 | ZNF783 | ZNF668 | POU5F1 | ZNF257 | FOXF1 |
| ZNF75D | HIVEP1 | HOXD1 | ZNF573 | C11orf95 | DMRT1 |
| PIAS2 | MGA | ASCL1 | ZNF350 | ZNF98 | ZNF518B |
| FOXJ1 | ZNF560 | RCOR3 | ZNF324 | PPARD | ZNF436 |
| GRHL1 | MSX2 | ZNF790 | ZBTB32 | ZNF223 | NHLH1 |
| KLF11 | ZNF174 | ZNF605 | ZNF394 | ZNF561 | ZBTB33 |
| ATF2 | TRPS1 | POU4F3 | FOXD4L5 | CDX1 | SP8 |
| NFAT5 | ZNF616 | ESRRA | AHRR | ZBTB3 | NR1D2 |
| MXD1 | DNAJC2 | GLIS3 | PRDM4 | TADA2B | FLI1 |
| SMAD3 | ATF3 | ZNF519 | GFI1B | LHX2 | REPIN1 |
| DMRT2 | HOXB3 | ZNF354A | ZNF341 | AC005324.4 | SNAI2 |
| BARHL1 | ZNF101 | ZNF214 | KLF15 | MEIS2 | TFAP2A |
| IKZF4 | ZNF583 | AC019117.3 | ZMIZ1 | MLXIPL | ETS2 |
| ZNF607 | ZNF880 | ZFAT | ZSCAN4 | ZNF274 | ZNF735 |
| E2F7 | ELF4 | ZKSCAN4 | ZNF850 | HOXB2 | HOXC5 |
| ZNF595 | ZHX2 | AC008770.2 | ZNF256 | ZNF816 | SOX9 |
| ETV5 | GSC | FOXA1 | ZBTB46 | GTF3A | NR2C1 |
| CSRNP3 | ZNF71 | ZNF658 | HSFY1 | NKX2-4 | NFYA |
| CEBPD | HBP1 | PITX3 | HSFY2 | ZNF134 | NKX3-2 |
| ZNF669 | HOXB8 | ZNF496 | HOXC8 | ETV2 | TTF1 |
| TBX22 | ZNF302 | ZNF248 | ZNF33A | POU2F1 | NR4A3 |
| ZNF304 | ZFP41 | KLF10 | ARNTL | SALL1 | ZNF768 |
| FOXN4 | TBX20 | BCL6 | RLF | ZNF136 | SETDB2 |
| TBX6 | NEUROG1 | HKR1 | SOX1 | SP140L | FOXI1 |
| ZBED2 | DUX4 | TCF7L2 | DMRT3 | ZNF780B | ZNF574 |
| HES5 | PKNOX2 | MAEL | ZNF624 | IRF5 | FOXL1 |
| ZNF283 | SMARCA1 | NR1H4 | NPAS4 | ZNF443 | ZNF527 |
| PLAG1 | ZSCAN18 | POU5F1B | ZSCAN30 | ETV1 | CIC |
| ZNF512B | ZNF334 | ZNF316 | GLIS1 | HMG20B | HES6 |
| ZNF486 | ZNF219 | POU2F2 | ZNF146 | PGR | NKX2-6 |
| ZMIZ2 | THRA | ZNF432 | FOXM1 | MYBL2 | NR1D1 |
| TERF2 | ZIM2 | HSF5 | AC092329.3 | EGR2 | ZNF121 |
| TFAM | TCF24 | ZNF382 | SIX2 | ZNF397 | ZBTB7C |
| LCORL | ZNF181 | ZNF507 | KLF18 | NFATC2 | ZNF619 |
| THAP5 | ALX4 | MYCL | MESP1 | OTX1 | SOX2 |
| TCF15 | HDX | SNAI1 | BHLHE40 | CREM | ZNF131 |
| ZNF75A | ZNF530 | NKX6-3 | ZNF483 | FOXO3 | E2F1 |
| HMGB2 | GMEB2 | SREBF2 | HES3 | ZBTB6 | ZNF678 |
| EGR3 | SMAD9 | ZNF347 | RUNX1 | ZNF433 | ZSCAN25 |
| ZFP69B | HOXC11 | ZNF281 | ZNF430 | CREB1 | THAP8 |
| USF1 | ZNF502 | BNC2 | MYNN | POU3F4 | ZNF596 |
| TBR1 | ZNF114 | HOXA10 | HSFX4 | ZNF672 | ZNF622 |
| SMAD2 | TBX4 | SOX4 | ZNF711 | ZNF329 | ZBTB2 |
| ZNF165 | E2F6 | SPI1 | TBX5 | SCRT1 | ZSCAN26 |
| HOXA11 | FOXD4L4 | ZFP90 | POU6F2 | ZNF140 | MYF6 |
| LMX1A | ZNF233 | ZNF23 | RXRA | ZBED6 | ZNF236 |
| LHX6 | ZNF230 | SOX11 | WDHD1 | ZNF765 | ZNF589 |
| ZNF155 | ZNF594 | MIS18BP1 | LBX2 | CRX | TWIST2 |
| ZNF16 | TULP1 | HLX | ZNF764 | ZNF853 | PATZ1 |
| ZNF225 | HOXB9 | ZNF85 | RFX6 | BAZ2B | LBX1 |
| ZSCAN10 | SSRP1 | ZNF799 | SOX17 | ZNF182 | ZBTB40 |
| HNF1B | DLX3 | MEF2C | ZFP91-CNTF | ZNF467 | ZSCAN16 |
| AC008758.1 | ZBTB1 | PRDM16 | ZHX1 | ZNF253 | ZNF202 |
| HOXA13 | AHR | ZNF408 | ZNF48 | PRDM10 | ZNF782 |
| MKX | ZNF660 | ZNF200 | SP7 | ZNF333 | HSFX3 |
| TBX1 | CSRNP2 | ZNF883 | CSRNP1 | PRDM1 | ZNF721 |
| ZNF70 | HIVEP3 | ZNF526 | DLX6 | ZIM3 | ZNF676 |
| ZNF282 | FOXJ3 | DBP | HSF1 | GATA4 | ZXDC |
| PRDM13 | PRDM8 | ZNF473 | ERG | GLIS2 | ZNF576 |
| ZNF786 | RORC | ZNF551 | MAF | ZNF180 | POU3F1 |
| ZNF547 | CEBPG | IKZF2 | ZNF404 | ZNF366 | IRF6 |
| EOMES | BACH1 | E4F1 | AL033529.1 | SOX18 | OSR1 |
| HOXB1 | ZNF705B | OTP | ZNF275 | ZFP1 | PRDM14 |
| ZKSCAN3 | ZNF319 | ZNF800 | RFX3 | ZNF517 | ZNF586 |
| ZNF648 | FOXR1 | FERD3L | MAZ | HOXD10 | TBX19 |
| MECP2 | ZNF41 | ZNF865 | IKZF5 | ZNF112 | FOXN2 |
| WIZ | YBX1 | ZNF707 | KLF9 | CCDC88A | EVX2 |
| ZNF317 | SATB2 | ZNF606 | ZNF665 | ZNF628 | ZBED1 |
| ZGLP1 | ZSCAN1 | SOX8 | ZNF682 | ZNF829 | IRX6 |
| BACH2 | ZNF571 | GCM1 | ZNF566 | TADA2A | PBX2 |
| IRX4 | NKX2-8 | BCL6B | FOXB1 | ASCL3 | CLOCK |
| IRF4 | ZNF3 | HEYL | DBX2 | ZNF688 | TAL1 |
| HOXA9 | TEAD2 | SALL4 | FOXG1 | ZNF784 | HELT |
| SMAD7 | NR5A1 | ZNF18 | JUNB | MYOD1 | DLX5 |
| ZNF613 | ZNF485 | ZSCAN5B | DPRX | SMAD5 | GTF2IRD2 |
| SOX30 | NR2F2 | MTA1 | MAFF | GSX1 | HMGB1 |
| TSC22D4 | ZNF491 | NR6A1 | SRY | DMBX1 | HMX1 |
| MEOX1 | SP6 | JUN | FOXP4 | ZNF419 | GBX2 |
| ZNF438 | ZNF439 | ZNF141 | MYRF | SP110 | NFIX |
| TCF4 | KLF14 | NFX1 | AFF2 | SALL3 | ZNF646 |
| ZBTB18 | IKZF1 | ARX | OVOL2 | BBX | ZNF479 |
| RBPJL | UBP1 | PEG3 | TGIF2LY | FEZF2 | AC073111.3 |
| LYL1 | DUXB | RARB | ZSCAN29 | EBF2 | EVX1 |
| BHLHE22 | FOXA3 | NCOR2 | ZNF852 | HMGA2 | MZF1 |
| AC010422.6 | ZBTB26 | UBTFL1 | TCF23 | DACH1 | RUNX3 |
| IRF3 | ZNF215 | ZNF331 | FOSB | SETDB1 | HOXD12 |
| ZNF260 | ZNF284 | ZNF778 | BHMG1 | VAX2 | JUND |
| PROX2 | ZNF667 | NRL | NKX2-2 | MEF2D | RXRB |
| TP63 | ZKSCAN2 | MYOG | POU3F2 | ZNF273 | ZNF208 |
| HOXA6 | THAP9 | NOTO | NR2F6 | ZNF287 | ZNF383 |
| DMRTB1 | TCF21 | ZNF205 | BCL11A | ZNF37A | ESRRB |
| CCDC169-SOHLH2 | ZNF197 | LEF1 | AC115220.1 | RAX | ZNF891 |
| YY2 | ZNF549 | ZNF529 | ZNF268 | OSR2 | ZNF699 |
| ZNF143 | PROX1 | HOXA2 | TULP4 | ZNF763 | NR2E3 |
| PBRM1 | TCFL5 | ZNF148 | ASCL5 | TSHZ3 | ZNF335 |
| ZNF841 | FOSL2 | SMARCC1 | HIC1 | ZNF555 | STAT1 |
| ADNP | E2F3 | ZNF322 | ZBTB45 | HOXC10 | GATAD2A |
| SPIB | BAZ2A | TAL2 | ZNF836 | TOX | ZFPM1 |
| BCL11B | HMGB3 | EMX2 | ZNF670 | KLF8 | NFE2L2 |
| ZNF513 | ZNF599 | KLF2 | ZFHX2 | ZNF235 | ZNF276 |
| NEUROD6 | ZNF492 | ZBTB22 | FOXF2 | ZNF746 | ESRRG |
| SRF | ZNF232 | ZNF550 | SMARCE1 | VAX1 | ZNF835 |
| TBXT | MIER3 | ZFP3 | ZNF789 | BARX2 | TP53 |
| HOXD9 | GLI1 | ZNF597 | MYT1L | ZNF311 | ZNF514 |
| ZBTB47 | ZBTB24 | PBX4 | ZNF24 | RBPJ | ZFP82 |
| ZNF22 | ZNF532 | ZNF652 | TBX3 | PDX1 | ZNF728 |
| NFXL1 | ZNF644 | CSDC2 | NEUROD4 | ZXDB | PBX1 |
| ZNF543 | HOXD3 | TPRX1 | AR | ZNF528 | NR1I3 |
| ZNF540 | UBTF | ZFHX3 | MBD2 | BATF3 | ZNF780A |
| SOX14 | ZNF410 | ZNF358 | ZNF189 | HOXC12 | NPAS2 |
| TFAP4 | SP140 | FOXD1 | PHOX2A | SMAD1 | ZNF154 |
| ASCL2 | ZNF92 | STAT2 | ZFP64 | ZFP91 | RERE |
| HEY1 | HINFP | ZNF431 | SMAD6 | HMX2 | NFATC1 |
| EBF3 | LHX8 | SREBF1 | THAP10 | ZNF416 | SALL2 |
| XBP1 | AC025287.4 | ZNF69 | AC073111.5 | ZNF776 | ATF6 |
| ZNF562 | ZNF20 | FOXB2 | FOXD4L3 | SPDEF | PBX3 |
| PROP1 | ZNF418 | MIER1 | TP73 | ARID3C | JARID2 |
| CDX4 | ZBTB14 | LRRFIP2 | ZNF587B | ZNF417 | PKNOX1 |
| SOX10 | MLXIP | GATA6 | HOXB13 | FOXO1 | ELF5 |
| BORCS8-MEF2B | CREB3L4 | HIVEP2 | BHLHA9 | ZNF709 | ZNF714 |
| ZNF211 | SCX | ZNF100 | ZNF184 | FOXH1 | NFIB |
| ZNF266 | NANOGP8 | SOHLH2 | ZNF471 | ZNF80 | NR0B1 |
| ZNF580 | ZNF548 | ZKSCAN8 | HOXA5 | ZNF296 | ZNF664 |
| OVOL3 | L3MBTL4 | HOXC9 | ZNF362 | ZFX | DACH2 |
| ZNF391 | ZNF655 | SP9 | ZBTB17 | SP4 | SOX15 |
| ATOH1 | AC118549 | ZNF684 | ZNF160 | ID1 | RBAK |
| ATF5 | TBX10 | ZNF484 | GABPA | ZNF701 | ZNF14 |
| MYB | ZNF587 | ZNF570 | ZNF142 | CEBPB | CDIP1 |
| ALX3 | AC012531.3 | ZNF251 | ZNF771 | ZNF66 | VSX1 |
| SP2 | ELF1 | NR1H3 | THAP12 | TULP2 | HOXD13 |
| THAP2 | AC073612.1 | ZIC3 | HMX3 | RFX1 | ZNF423 |
| GATA2 | LIN28A | FEV | ZNF736 | ZNF724 | ZNF552 |
| CDX2 | HMGXB4 | NFYB | ZFP14 | GRHL2 | ZNF730 |
| OLIG2 | ZNF396 | ZNF677 | RORA | ZNF879 | ZNF781 |
| SIM1 | NR2E1 | HOPX | PAX4 | TEAD3 | AC010616.1 |
| ZNF445 | ZNF823 | ZNF460 | ZBTB4 | ZNF541 | IRX1 |
| YBX2 | STAT4 | ARID3B | HOXC4 | ZNF610 | ARID1A |
| ZBTB10 | ZNF626 | ZNF581 | CREB5 | ELK4 | ZNF716 |
| ZNF280B | ZNF470 | POU4F1 | TEAD4 | YY1 | CEBPZ |
| GLMP | HHEX | KLF12 | ZNF250 | ZNF76 | PLEK |
| ZNF649 | ANHX | ZNF345 | FOXD2 | AC020909.1 | EPAS1 |
| NKX1-1 | L3MBTL1 | TEAD1 | JAZF1 | ZSCAN2 | SFPQ |
| ZNF469 | ASCL4 | LMX1B | MYRFL | ZNF12 | GON4L |
| VEZF1 | ZNF480 | PIAS1 | THAP1 | DNAJC1 | PHTF1 |
| ZNF518A | FOXI3 | NR1H2 | ZNF578 | ZNF585A | BLZF1 |
| TFDP1 | ZFP57 | ZNF324B | ZNF461 | ZNF385B | ZNF706 |
| RELA | RFX8 | MYCN | IKZF3 | FOXA2 | KIAA1549 |
| CREB3L3 | GLI4 | ZNF175 | FOXJ2 | ZSCAN9 | ZNFX1 |
| GZF1 | MBD3 | VENTX | AC187653.1 | ZNF814 | SLC2A4RG |
| ZNF19 | ZNF732 | ZNF559-ZNF177 | ZNF788 | ZNF697 | RBCK1 |
| SAMD11 | TCF12 | VDR | ZNF708 | ZNF462 | YEATS4 |
| RUNX2 | ELK1 | ZNF546 | TERB1 | ZNF618 | CASZ1 |
| HSF2 | DBX1 | AFF3 | ZFP92 | CREB3L2 | CC2D1A |
| CARHSP1 | GFI1 | MBD1 | IRF8 | ZNF827 | ZNF414 |
| TFCP2L1 | ZNF726 | KLF16 | ZNF705D | ZNF77 | XPA |
| TSHZ1 | SIX3 | ZNF737 | MXD3 | NEUROG2 | TCF19 |
| ZNF234 | ZSCAN12 | ZNF169 | ZNF506 | MSC | ZNF280D |
| ZNF43 | BATF2 | ZNF695 | RHOXF2 | NEUROD1 | PREB |
| HIF3A | NR5A2 | SP5 | NHLH2 | ZBTB12 | SMARCAL1 |
| FOXE3 | ZEB2 | CAMTA2 | STAT5B | ZNF629 | CARF |
| POU5F2 | GTF2I | SOX6 | ZNF490 | ATOH7 | TCF25 |
| NOBOX | ZNF568 | CAMTA1 | ZNF516 | KLF7 | ZNF750 |
| FOXR2 | ZNF217 | DDIT3 | NFKB2 | ZBTB48 | GABPB2 |
| ID4 | CDC5L | ZNF525 | ZNF99 | MESP2 | TMF1 |
| ZNF93 | TSC22D2 | LEUTX | ESR1 | FOXK1 | LYAR |
| PTF1A | PIAS3 | AC092835.1 | DMRTA2 | ZBTB41 | PURB |
| ZNF625 | IRX5 | ZKSCAN7 | INSM1 | NR2C2 | CIZ1 |
| PAX6 | ERF | ZNF285 | SOX5 | ZNF558 | ANKRD30A |
| ZNF267 | ZFP37 | RCOR2 | TBX18 | RFX4 | THYN1 |
| PAX7 | ETV3 | ARGFX | HNF4G | SMARCC2 | SETBP1 |
| BHLHE23 | TFE3 | ZNF30 | ATF1 | PLAGL1 | AHCTF1 |
| GRHL3 | CTCF | MNX1 | SCRT2 | ZBTB7B | ELMSAN1 |
| ZNF512 | EGR1 | MYF5 | NKX1-2 | ZNF425 | GATAD1 |
| ZFP2 | TFDP3 | ZNF354B | ZNF384 | HOXB4 | SON |
| ZNF787 | E2F2 | NKX3-1 | PLAGL2 | ZNF500 | GPBP1L1 |
| GMEB1 | ZFP36L2 | KLF3 | POU4F2 | FOXL2 | ZNF326 |
| ZNF700 | NFIC | FOXD4L1 | NANOG | PHOX2B | SPZ1 |
| SIX5 | LCOR | PITX2 | ZNF81 | ISX | FAM170A |
| HOXD8 | ZNF614 | ZNF772 | ZNF497 | ZBTB21 | CREBRF |
| EBF4 | RFX7 | ZBTB16 | NR0B2 | ZNF582 | ZNF704 |
| ZNF692 | ZBTB7A | ZNF575 | AC010487.3 | SP100 | ZNF503 |
| MYT1 | ZNF717 | ZNF620 | AC004080.3 | ZSCAN21 | RAG1 |
| CPHXL | ZNF34 | ZNF761 | ZNF135 | STAT6 | PHB |
| ZNF615 | NR4A2 | ZNF793 | RELB | FOXC2 | TET2 |
| NR2F1 | MEF2A | ZNF254 | GTF2IRD1 | TCF3 | ZNF608 |
| IRF7 | ZNF367 | TFAP2B | CUX1 | LHX1 | ZNF280A |
| ZNF117 | RCOR1 | SNAPC4 | ZNF207 | ID3 | CENPX |
| ZNF579 | ZNF264 | ZNF675 | LTF | ARID5A | CNBP |
| ZNF556 | ZNF441 | ZNF860 | PRDM11 | PAX3 | NFRKB |
| ZNF777 | TWIST1 | FOXD3 | BCLAF1 | DEAF1 | ZNF318 |
| FOXE1 | AFF4 | ZNF132 | TFB1M | ZNF44 | PURG |
| ZNF680 | STAT5A | NFE2L3 | ZNF280C | KLF1 | CENPS |
| ZIC4 | ZNF888 | ZNF510 | PARP12 | GATAD2B | MAF1 |
| NFYC | AC023509.3 | HOXC6 | GPBP1 | SIX1 | ZNF609 |
| PRDM15 | ZNF805 | THRB | RFXANK | ONECUT2 | DMRTC1B |
| ZNF468 | ZHX3 | ZNF774 | MLLT10 | ZFY | PURA |
| ID2 | ZNF584 | ZFP28 | KHSRP | ATMIN | NKRF |
| ZSCAN5A | PRRX1 | NFE2L1 | HIF1A | NFIA | ZNF395 |
| ISL2 | HOXA3 | HOXD4 | ADNP2 | FOXI2 | PLSCR1 |
| ZNF710 | E2F5 | ZNF681 | CENPT | TFCP2 | ZGPAT |
| ZIC5 | KLF17 | NFIL3 | CAPN15 | MBD4 | ZNF720 |
| ZBED4 | RHOXF1 | FOXP3 | GABPB1 | ZIC1 | ZNF511 |
| ZNF679 | NKX2-3 | CUX2 | MYEF2 | POU6F1 | L3MBTL3 |
| HMBOX1 | ZNF691 | CREB3 | TAX1BP1 | FIGLA | CPEB1 |
| RFX5 | ZSCAN31 | ARNT2 | AKNA | TFAP2E | ZNF487 |
| ZNF687 | ZNF337 | MTA3 | CREBL2 | ZNF213 | NME2 |
| ZNF773 | ZNF429 | AEBP2 | CENPA | ZNF17 | ZNF488 |
| HOXB7 | HAND1 | DMRTC1 |  |  |  |
